# Hepatocyte‐Derived Extracellular Vesicles Deliver miR‐328‐3p to Trigger PP2A‐B56δ–Mediated p‐NLRP3^S295^‐Dependent Metaflammation in Macrophages upon Microcystin‐LR Exposure

**DOI:** 10.1002/advs.202507039

**Published:** 2025-11-19

**Authors:** Jia‐Shen Wu, Xin‐Yu Zhang, Xin‐Yu Ma, Yue‐Yue Wei, Lei‐Lei Wang, Ze‐Bang Du, Xiao‐Gang Xia, Lin Che, Dong‐Bei Guo, Han‐Ying Zheng, You‐Liang Yao, Wen‐Gang Li, Yu‐Chun Lin, Zhong‐Ning Lin

**Affiliations:** ^1^ State Key Laboratory of Vaccines for Infectious Diseases Xiang An Biomedicine Laboratory，Xiang'an Hospital of Xiamen University National Innovation Platform for Industry‐Education Integration in Vaccine Research School of Public Health Xiamen University Xiamen 361102 China; ^2^ Department of Hepatobiliary Surgery Cancer Research Center Xiang'an Hospital of Xiamen University School of Medicine Xiamen University Xiamen Fujian 361102 China; ^3^ State Key Laboratory of Oncology in South China Guangdong Provincial Clinical Research Center for Cancer Sun Yat‐sen University Cancer Center Guangzhou 510060 China

**Keywords:** Extracellular vesicle miR‐328‐3p, MC‐LR‐associated MASLD, p‐NLRP3^S295^‐dependent metaflammation, PP2A‐B56δ, Site‐specific monoclonal antibody

## Abstract

Microcystin‐LR (MC‐LR) exacerbates metabolic dysfunction‐associated steatotic liver disease (MASLD) by inducing histopathological damage and lipid metabolism disorders. Inducible hepatocyte‐derived extracellular vesicles (iHD‐EVs) released after xenobiotic exposure activate the macrophage NOD‐like receptor protein 3 (NLRP3) inflammasome. Suppression of NLRP3 phosphorylation at serine 295 (p‐NLRP3^S295^) is previously shown to alleviate MASLD progression. Here, it is demonstrated that microcystin‐LR (MC‐LR)‐induced iHD‐EVs reduced deliver of miR‐328‐3p to macrophages, thereby upregulating protein phosphatase 2A (PP2A)‐B56δ. Consequent PP2A‐B56δ activation disrupts inositol 1,4,5‐triphosphate receptor and voltage‐dependent anion channel 1 coupling, evokes mitochondria‐associated endoplasmic reticulum membrane (MAM) calcium (Ca^2+^) overload, and recruits p‐NLRP3^S295^ into the inflammasome. Neutralization of p‐NLRP3^S295^ with a site‐specific monoclonal antibody (anti‐p‐NLRP3^S295^ mAb) markedly attenuates liver inflammation and injury in MC‐LR‐exposed mice. Collectively, the miR‐328‐3p/PP2A‐B56δ/p‐NLRP3^S295^ axis is identified as a crucial driver of metaflammation and establishes circulating EV‐miR‐328‐3p as a novel biomarker and anti‐p‐NLRP3^S295^ mAbs as translational tools for MC‐LR‐associated MASLD.

## Graphical abstract

Hepatocyte‐derived extracellular vesicles deliver miR‐328‐3p to trigger PP2A‐B56δ–mediated p‐NLRP3^S295^‐dependent metaflammation in macrophages upon microcystin‐LR exposure. 2‐DG, 2‐deoxy‐D‐glucose. B56δ, protein phosphatase 2A‐B56δ. ER, endoplasmic reticulum. iHD‐EV, inducible hepatocyte‐derived extracellular vesicles. IP3R, inositol 1,4,5‐triphosphate receptor type 3. GSDMD, Gasdermin D. HC, hepatocyte. mAb, monoclonal antibody. MC, macrophage. MAM, mitochondria‐associated endoplasmic reticulum membrane. MASLD, metabolic dysfunction‐associated steatotic liver disease. MC‐LR, microcystin‐leucine arginine. Mito, mitochondrion. NLRP3, NOD‐like receptor protein 3. Nu, nucleus. p‐NLRP3^S295^, phosphorylation of NLRP3 at serine 295. VDAC1, voltage‐dependent anion channel 1.

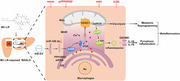

## 1 Introduction

Metabolic dysfunction‐associated steatotic liver disease (MASLD) affects ≈25 % of the global population and is tightly linked to obesity, dyslipidemia, hypertension, and insulin resistance.^[^ ^1^ ^]^ Xenobiotic exposure accelerates the transition from simple steatosis to metabolic dysfunction‐associated steatohepatitis (MASH) by aggravating lipid metabolism disorders and fostering a pro‐inflammatory microenvironment. Among contaminants of emerging concern (CECs), microcystin‐LR (MC‐LR) is a potent hepatotoxin and carcinogen detected in polluted water and food worldwide.^[^ ^2^, ^3^ ^]^ Althoguh the World Health Organization (WHO) guideline limits MC‐LR in drinking water to 1 µg L^−1^, fish from Lake Taihu have been measured at 2.64 µg kg^−1^, surpassing the WHO tolerable daily intake.^[^ ^4^, ^5^ ^]^ MC‐LR exposure disrupts intestinal permeability and induces NOD‐like receptor protein 3 (NLRP3)‐dependent immunotoxicity in the liver.^[^ ^6^ ^]^ Long‐term low‐dose MC‐LR exposure promotes hepatocyte inflammation and lipid metabolism disorders through gut dysbiosis and NLRP3 inflammasome activation.^[^ ^7^ ^]^ Administration of 120 µg kg^−1^ MC‐LR to mice or exposure of primary human hepatocytes to 0.005–1 µM MC‐LR induces marked toxicity.^[^ ^8^ ^]^ MC‐LR hepatotoxicity recruits macrophages in a dose‐dependent manner, highlighting the importance of hepatocyte‐macrophage intercellular communication in liver inflammatory injury.^[^ ^9^ ^]^ Evidence indicates that hepatic macrophages increase lipid uptake and transition it to a pro‐inflammatory phenotype to promote hepatitis upon MC‐LR exposure, indicating metabolic reprogramming in activated macrophages.^[^ ^10^ ^]^ These findings imply the need to clarify macrophage activation and function during MC‐LR‐exposed MASLD progression and identify novel biomarkers of macrophage inflammatory regulation for the treatment of MASLD.

Extracellular vesicles (EVs) mediate intercellular communication via selectively packaged cargo and long‐range delivery. In MASLD, injured hepatocytes release EVs that orchestrate liver inflammation; yet hepatocyte–macrophage EV crosstalk remains under‐explored. Hepatocyte‐derived EVs enriched in DNA, RNA, polypeptides, and lipids modulate hepatic insulin resistance, macrophage pro‐inflammatory polarization, and fibrosis, thereby accelerating MASLD progression.^[^ ^11^, ^12^, ^13^ ^]^ Under lipotoxic stress or hypoxia, hepatocytes secrete pro‐inflammatory EVs that activate Kupffer cells, highlighting the functional significance of EVs cargo.^[^ ^14^, ^15^ ^]^ MicroRNAs (miRNAs) transported by EV orchestrate distinct stages of MASLD.^[^ ^16^ ^]^ For example, miR‐192‐5p promotes macrophage inflammation by activating the Rictor–Akt–FoxO1 axis, whereas loss of miR‐690 reduces hepatic macrophage inflammation.^[^ ^17^ ^]^ Oxidized LDL disrupts lysosomal cholesterol trafficking, enhances hepatocyte EV release, and drives M1 macrophage polarization via miR‐122‐5p.^[^ ^18^ ^]^ These findings identify EV‐miRNAs as key regulators of macrophage activation and candidate early biomarkers of MASLD. We previously demonstrated that xenobiotic‐exposed hepatocyte‐derived EVs remodel the hepatic immune microenvironment by transcriptionally activating macrophages and driving MASLD progression. Although miRNAs regulate NLRP3 expression, activation, and inflammasome assembly,^[^ ^19^, ^20^ ^]^ the function of hepatocyte‐derived EV‐miRNAs in macrophage NLRP3‐associated metaflammation remains incompletely defined.

Targeted macrophage NLRP3 intervention alleviates xenobiotic‐driven hepatic inflammation and fibrosis, thereby limiting liver injury.^[^ ^21^, ^22^ ^]^ MC‐LR exposure–associated NLRP3 inflammasome activation has been implicated in fatty liver development and insulin resistance.^[^ ^23^ ^]^ Damage‐associated molecular patterns (DAMPs) encompass endoplasmic reticulum (ER) Ca^2+^ dyshomeostasis, NLRP3 priming, pyroptotic execution, and pro‐inflammatory cytokine release.^[^ ^24^ ^]^ Post‐translational modifications (PTMs) are essential for NLRP3 activation. Specifically, the sustained phosphorylation at serine 295 (p‐NLRP3^S295^) governs NLRP3 translocation and dissociation from the mitochondria‐associated ER membrane (MAM), representing a key activation checkpoint.^[^ ^25^ ^]^ Protein phosphatase 2A (PP2A) dephosphorylates NLRP3 at serine 5, modulating protein oligomerization and inflammasome assembly and underscoring the regulatory role of NLRP3 phosphorylation in MASLD therapy.^[^ ^26^ ^]^ Currently, no drugs are approved for MASLD; candidates targeting energy intake, metabolism, and inflammation remain under development. Emerging anti‐inflammatory biologics, such as OKT3 and CD3 monoclonal antibodies (mAbs), show promise.^[^ ^27^, ^28^ ^]^ To strengthen mAb specificity, intracellular neutralizing antibodies (intrabodies), including single‐chain Fv fragments and nanobodies, are being engineered for precise recognition of PTMs within intracellular proteins.^[^ ^29^ ^]^ Consequently, development of site‐specific anti‐p‐NLRP3^S295^ antibodies holds significant therapeutic potential for early intervention in MASLD.

In this study, employing in vitro (0.05 µM MC‐LR, 24 h) and in vivo (100 µg kg^−1^ body weight, i.g., 4 weeks) MC‐LR exposure models, we delineated how inducible hepatocyte‐derived EVs (iHD‐EVs) instigate macrophage p‐NLRP3^S295^‐dependent metaflammation and demonstrated that a custom anti‐p‐NLRP3^S295^ mAb attenuates inflammation and liver injury, offering a novel strategy against xenobiotic‐associated MASLD progression.

## 2 Results

### 2.1 Macrophage p‐NLRP3^S295^ Activation Drives MASLD Triggered by MC‐LR Exposure

**Figure 1:**
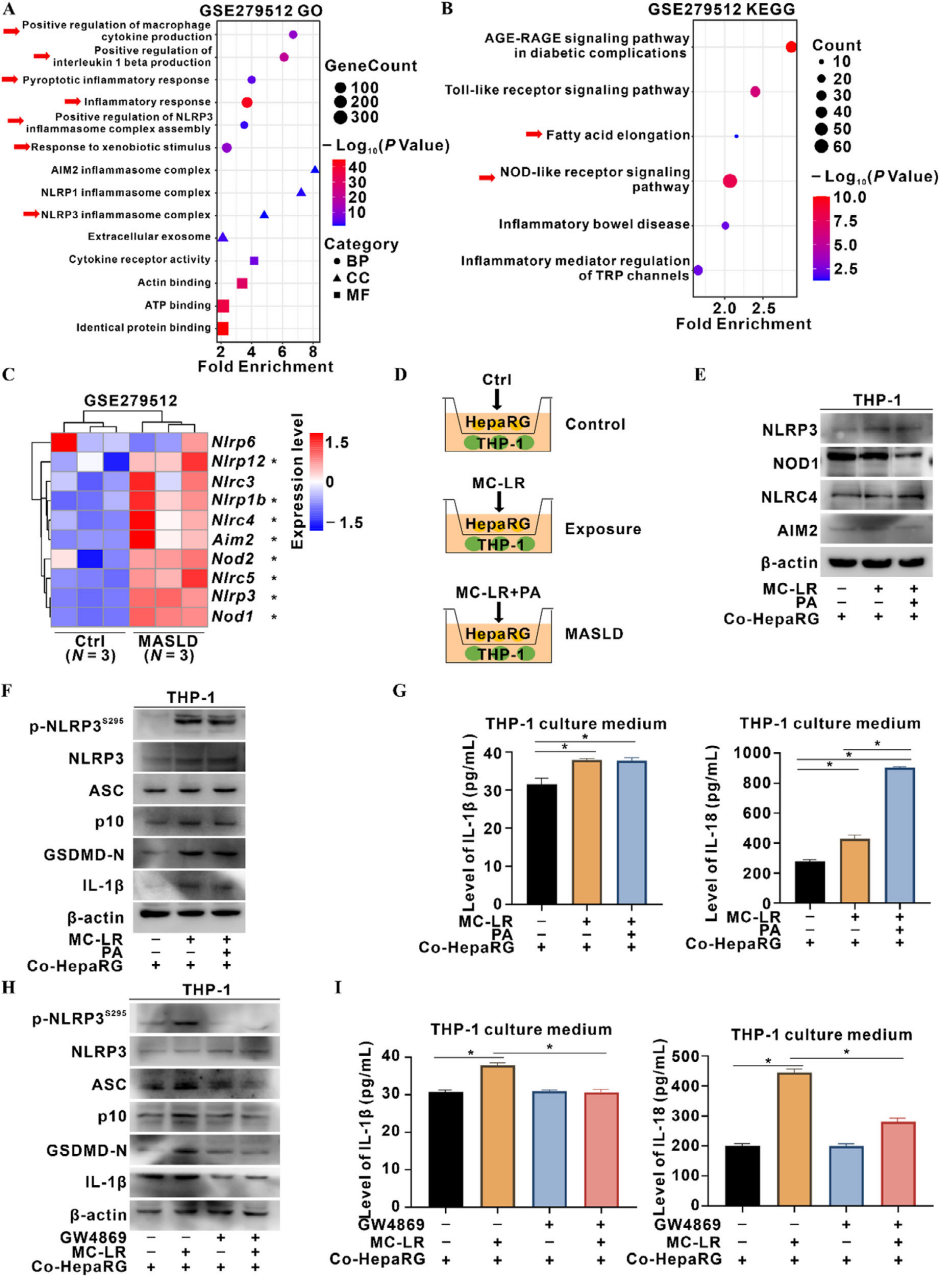
Macrophage p‐NLRP3^S295^ activation drives MASLD triggered by MC‐LR exposure. A–C) Liver transcriptomes from MASLD mice (GSE279512) were screened; mice received either chow diet (control) or high‐fat diet (HFD) plus streptozotocin (i.p., 60 mg kg^−1^ body‐weight) (MASLD group). *N* = 3 per group. DEGs were identified and subjected to GO A) and KEGG B) enrichment analyses. C) Heatmap displays inflammasome‐related mRNAs. D–I) A co‐culture model of HepaRG and THP‐1 macrophages was established in a Transwell system, as illustrated in the schematic diagram D). HepaRG cells were treated for 24 h with DMSO as the control (Ctrl) group, MC‐LR (0.05 µM) as the exposure group, or combined with MC‐LR and PA (200 µM) as the MASLD group E–G). E) Levels of NOD‐like receptors family members in co‐cultured THP‐1 cells were detected by WB. F) Levels of p‐NLRP3^S295^ and inflammasome proteins in co‐cultured THP‐1 cells were detected by WB. G) IL‐1β (Left) and IL‐18 (Right) levels in co‐cultured THP‐1 medium were quantified by ELISA. *N* = 3. H,I) HepaRG cells were pretreated with or without the EV‐release inhibitor GW4869 (10 µM, 24 h) prior to co‐culture with MC‐LR (0.05 µM, 24 h) exposure. H) Levels of p‐NLRP3^S295^ and inflammasome proteins in co‐cultured THP‐1 cells were detected by WB. I) IL‐1β (Left) and IL‐18 (Right) levels in co‐cultured THP‐1 medium were quantified by ELISA. *N* = 3. Data are presented as mean ± SD. *, *p* < 0.05, compared to the control or corresponding group.

MASLD progression is fueled by multiple insults, among which hepatic inflammation—elicited by either lipid accumulation or xenobiotic exposure—constitutes the dominant driver. Gene Ontology (GO) enrichment of differentially expressed genes (DEGs) in MC‐LR‐exposed HepaRG cells highlighted “protein phosphatase 2A binding”, “positive regulation of apoptotic process”, “negative regulation of miRNA transcription”, “extracellular exosome”, and “positive regulation of monocyte aggregation” pathways (Figure S1A, Supporting Information). These alterations indicate that MC‐LR‐induced hepatocellular injury promotes macrophage recruitment and activation, suggesting a hepatocyte–macrophage communication axis in MC‐LR‐associated liver injury. Parallel GO and KEGG analyses of DEGs in the liver transcriptomes from MASLD mice (GSE279512) revealed significant enrichment of “inflammasome assembly”, “pyroptotic inflammatory response”, “positive regulation of macrophage cytokine production”, and “NOD‐like receptor signaling” pathway (**Figure** **1**A–C). Consistently, inflammasome‐related transcripts were markedly upregulated, implying that NOD‐like receptor inflammasomes participate in MASLD progression. To extend these findings to human disease, we analyzed an RNA‐sequencing dataset derived from lipotoxicity‐challenged human precision‐cut liver slices. Significant concordance was observed among pathways activated in the mouse MASLD dataset, the HepaRG cell model, and the human liver slices, including “negative regulation of xenobiotic metabolic process”, “miRNA transcription”, “extracellular exosome”, “NOD‐like receptor pathway”, “calcium‐mediated signaling”, “glycolysis/gluconeogenesis”, “macrophage differentiation”, “positive regulation of interleukin‐1 beta production”, “inflammatory response”, “cytokine‐mediated signaling pathway”, and “triglyceride homeostasis” (Figure S1B,C, Supporting Information). Collectively, these results indicate that intercellular communication between hepatocytes and macrophages promotes inflammatory injury in MC‐LR‐associated MASLD.

Previous studies have demonstrated that long‐term exposure to 0.1 µM MC‐LR provokes hepatocyte damage, whereas short‐term exposure for 24 h is already sufficient to cause impairment, indicating that hepatotoxicity even at low doses.^[^ ^30^ ^]^ To define a sub‐lethal exposure window for the present study, we determined HepaRG cell viability after 24 h challenge with graded MC‐LR doses, yielding an IC_50_ of 2.225 µM (Figure S1D, Supporting Information). In line with earlier report and real‐time kinetic analyses (6–24 h), we employed 0.05 µM MC‐LR treatment in HepaRG cells to establish a sub‐cytotoxic hepatocyte injury model (Figure S1E, Supporting Information). To identify the NOD‐like receptor most responsive in MC‐LR‐associated MASLD, we constructed a Transwell co‐culture platform comprising HepaRG and THP‐1 cells. HepaRG monolayers were exposed to MC‐LR alone as the exposure group or in combination with palmitic acid (PA) to mimic MASLD conditions (Figure 1D). Lipid accumulation significantly increased in co‐cultured HepaRG cells in both MC‐LR exposure and MASLD groups (Figure S1F, Supporting Information). MC‐LR enters hepatocytes preferentially through organic anion‐transporting polypeptides (OATPs), leading to cell damage and apoptosis. The protein levels showed elevated BAX, Caspase‐3, and cleaved‐Caspase‐3 in co‐cultured HepaRG cells (Figure S1G, Supporting Information).

Profiling NOD‐like receptors in THP‐1 macrophages revealed selective up‐regulation of NLRP3 protein after co‐culture with MC‐LR‐exposed HepaRG cells, whereas NOD1, NLRC4, and AIM2 remained unchanged (Figure 1E). Concomitantly, transcript levels of *PRKD1*—encoding phosphokinase protein kinase D (PKD), the kinase for p‐NLRP3^S295^—increased in both MC‐LR exposure and MASLD groups (Figure S1H, Supporting Information). Activation of p‐NLRP3^S295^ and downstream inflammasome components were increased in THP‐1 cells, indicating initiation of p‐NLRP3^S295^‐dependent inflammation (Figure 1F). Moreover, we interrogated whether this inflammatory signature in co‐cultured THP‐1 cells is relayed through intercellular communication. The membrane co‐localization of GSDMD in THP‐1 cells co‐cultured with MC‐LR‐exposed hepatocytes was increased (Figure S1I,J, Supporting Information), paralleled by increased release of IL‐1β and IL‐18 (Figure 1G). To test the contribution of hepatocyte‐derived extracellular vesicles (HD‐EVs), we pretreated HepaRG cells with the EV‐release inhibitor GW4869 prior to co‐culture. GW4869 markedly blunted p‐NLRP3^S295^ activation in THP‐1 macrophages and decreased IL‐1β and IL‐18 release (Figure 1H,I). These results establish that HD‐EVs released from MC‐LR‐exposed hepatocytes are both necessary and sufficient to propagate p‐NLRP3^S295^‐dependent inflammasome activation in recipient macrophages, independent of direct MC‐LR actions on macrophages.

### 2.2 Inducible MC‐LR‐Exposed Hepatocyte‐Derived iHD‐EVs Trigger Macrophage p‐NLRP3^S295^‐Dependent Inflammation

**Figure 2:**
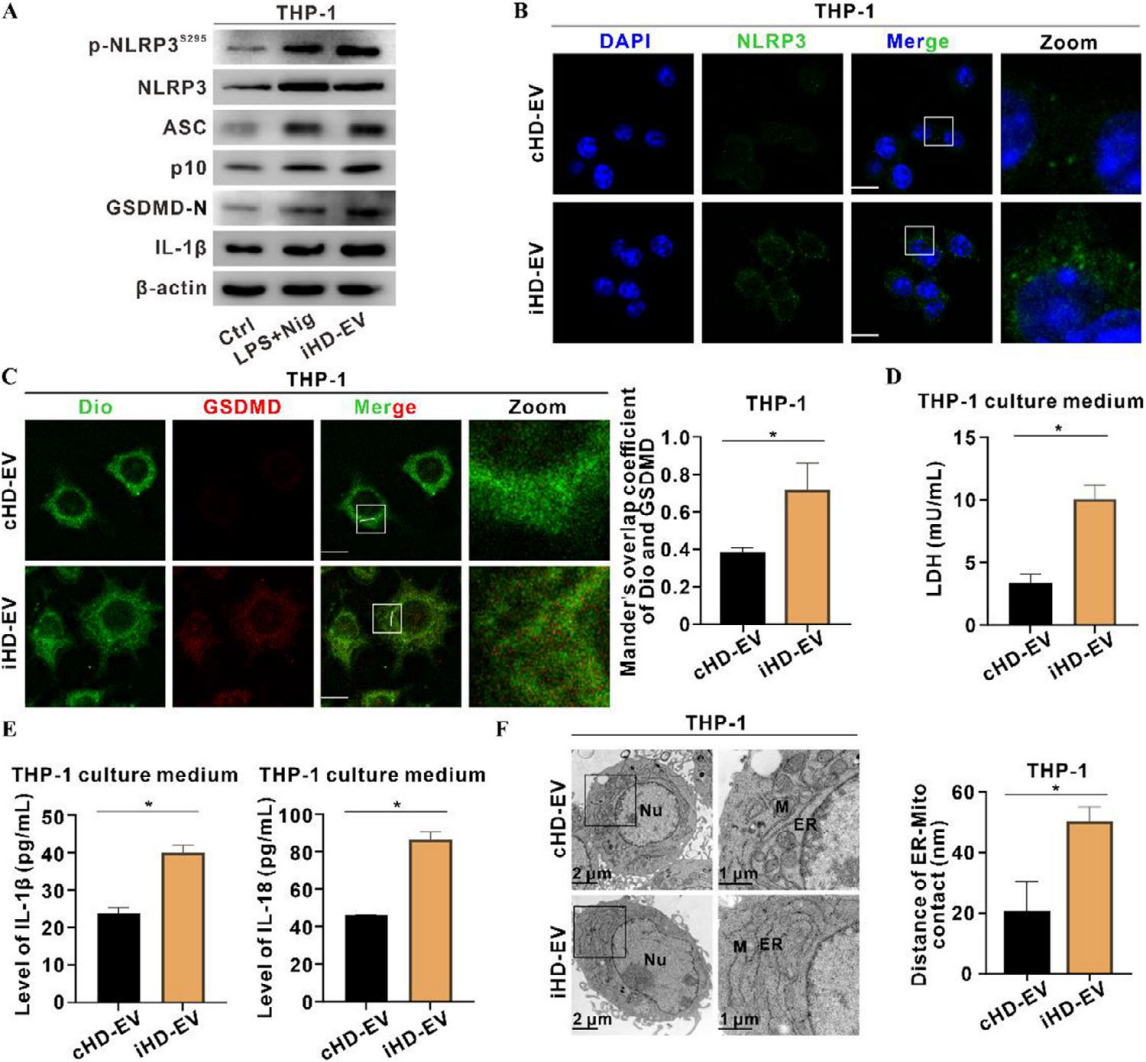
Inducible MC‐LR‐exposed hepatocyte‐derived extracellular vesicles (iHD‐EVs) trigger macrophage p‐NLRP3^S295^‐dependent inflammation. EVs were isolated from the culture medium of HepaRG cells exposed to 0.05 µM MC‐LR for 24 h as inducible hepatocyte‐derived EVs (iHD‐EV), while control hepatocyte‐derived EVs (cHD‐EV) were isolated from untreated HepaRG cells. THP‐1 cells were treated with cHD‐EV or iHD‐EV (40 µg mL^−1^) for 4 h. A) LPS (100 ng mL^−1^, 3 h) plus nigericin (Nig, 10 µM, 4 h) served as a positive control for NLRP3 activation. Levels of p‐NLRP3^S295^ and inflammasome proteins were detected by WB. (B) Representative IF images showing NLRP3 inflammasome specks (Green); nuclei were counterstained with DAPI (Blue). Scale bar, 10 µm. C) Representative IF images showing co‐localization of GSDMD (Red) with Dio‐stained membranes (Green) (Left). Scale bar, 10 µm. Manders' overlap coefficient is presented in the bar graph (Right). D) LDH release into the culture medium was measured. *N* = 3. E) IL‐1β (Left) and IL‐18 (Right) levels in culture medium were quantified by ELISA. *N* = 3. F) Representative TEM images showing MAM architecture in cells (Left). Quantification of ER–Mito distances is presented in the bar graph (Right). Scale bar, 2 µm (overview) and 1 µm (zoom). Data are presented as mean ± SD. *, *p* < 0.05, compared to the control or corresponding group.

To investigate the functional role of hepatocyte‐derived EVs, we isolated control EVs (cHD‐EVs) from untreated HepaRG cells and MC‐LR‐induced EVs (iHD‐EVs) from cells exposed to MC‐LR. These experiments were designed to dissect how MC‐LR‐evoked EV cargo propagates MASLD‐associated liver injury via EV‐mediated hepatocytes–macrophages communication. EVs were enriched by sequential ultracentrifugation and validated by transmission electron microscopy (TEM), nanoparticle tracking analysis, and immunoblotting for the EV markers CD9, CD81, TSG101, and CD63 (Figure S2A–C, Supporting Information). To track iHD‐EVs uptake in real time, HepaRG cells were transfected with pCDH‐CD63‐RFP, and RFP‐tagged EVs were added to THP‐1 macrophages. IF imaging showed greater accumulation of iHD‐EVs than cHD‐EVs in recipient macrophages (Figure S2D,E, Supporting Information). As a benchmark for canonical NLRP3 activation, macrophages were primed with lipopolysaccharide (LPS) and subsequently pulsed with nigericin (Nig) (Figure S2F, Supporting Information). Relative to cHD‐EVs, iHD‐EVs increased *PRKD1* mRNA levels (Figure S2G, Supporting Information) and elevated the levels of p‐NLRP3^S295^ and inflammasome proteins to an extent comparable to the LPS+Nig positive control (**Figure** **2**A).

To verify the necessity of Ser295 phosphorylation, we employed site‐directed mutagenesis to generate THP‐1 lines harboring NLRP3‐S295D (phospho‐mimetic) or NLRP3‐S295A (phospho‐null) mutants. iHD‐EV failed to augment NLRP3 inflammasome protein in S295A cell, whereas the response was preserved in S295D cells, confirming that phosphorylation at Ser295 is indispensable for iHD‐EV‐elicited signaling (Figure S2H, Supporting Information). THP‐1 cells stably expressing pB513B‐NLRP3‐GFP were imaged by live‐cell microscopy. iHD‐EVs triggered NLRP3 speck formation within 15 min (Figure S2I, Supporting Information). IF assay confirmed that iHD‐EVs induced NLRP3 foci formation (Figure 2B), promoted GSDMD translocation to membrane (Figure 2C; Figure S2J, Supporting Information), increased extracellular LDH, IL‐1β, and IL‐18 release (Figure 2D–E). TEM of iHD‐EVs‐treated macrophages revealed expanded ER–mitochondria contact sites and increased inter‐organellar gap distance, suggesting that iHD‐EV cargo perturbs ER–mitochondria tethering integrity in THP‐1 cells (Figure 2F). Collectively, these results establish that MC‐LR‐induced HD‐EVs are sufficient to propagate p‐NLRP3^S295^‐dependent inflammasome activation in macrophages, a process coupled to early disruption of ER–mitochondria homeostasis.

### 2.3 iHD‐EVs Trigger MAM Calcium Overload to License Macrophage p‐NLRP3^S295^‐Dependent Metaflammation

**Figure 3:**
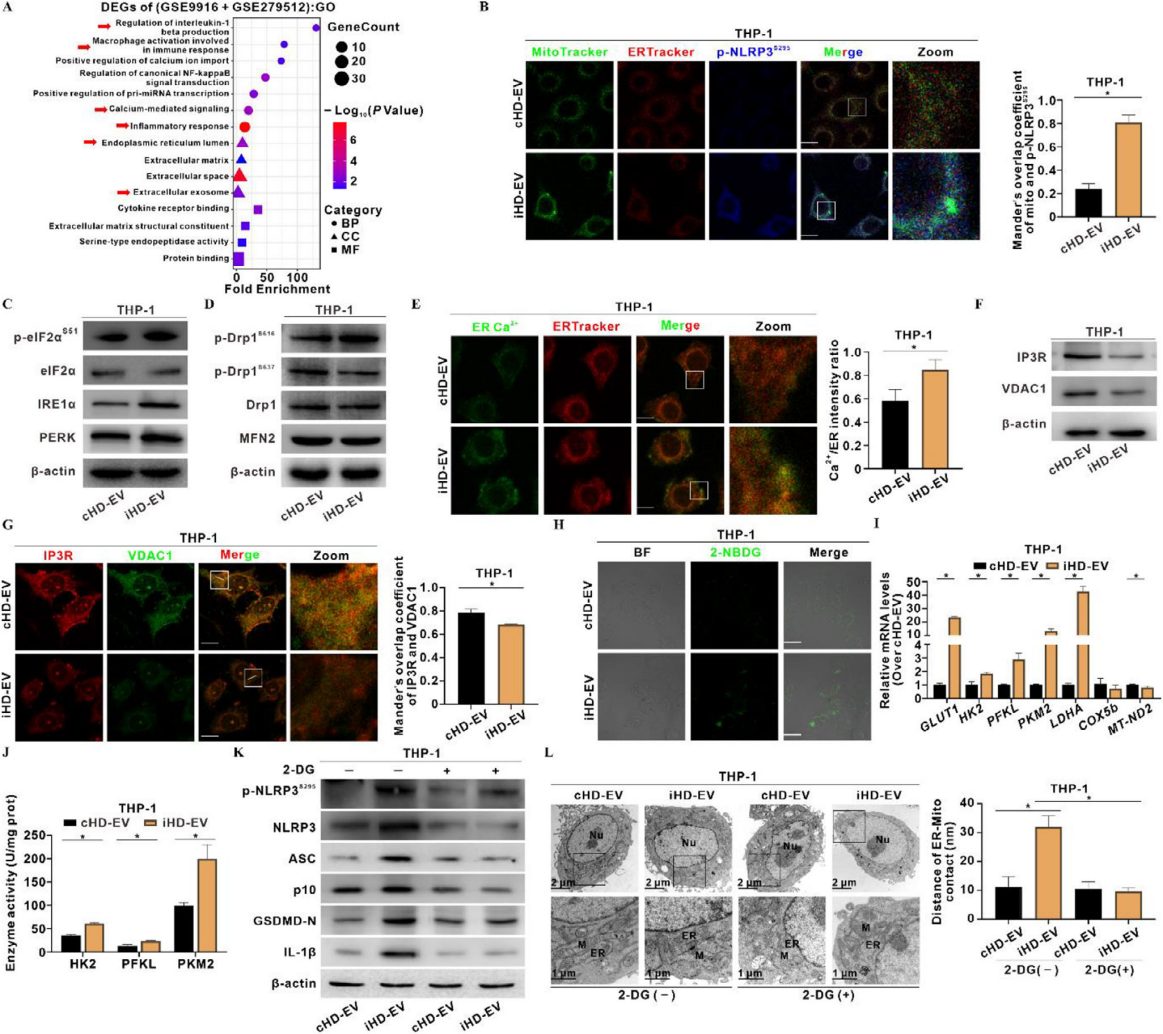
iHD‐EVs trigger MAM Ca^2+^ overload to license macrophage p‐NLRP3^S295^‐dependent metaflammation. A) Data retrieved from GEO databases, based on LPS‐activated human monocyte‐macrophages (GSE9916) and MASLD mouse liver transcriptomics (GSE279512), were mined for shared DEGs. GO analysis identified enriched signaling pathways among the shared DEGs. B–L) THP‐1 cells were treated with cHD‐EV or iHD‐EV (40 µg mL^−1^, 4 h). B) Representative IF images showing MAM localization of p‐NLRP3^S295^ (Blue) (Left); mitochondria and ER are labeled with MitoTracker (Green) and ERTracker (Red), respectively. Scale bar, 10 µm. Manders' overlap coefficient is shown in the bar graph (Right). C,D) Levels of ER stress C) and MQC‐related proteins D) were detected by WB. E) Representative IF images showing the level of ER Ca^2+^ (Fluo‐5N AM, Green) (Left); ER are labeled with ERTracker (Red). Scale bar, 10 µm. Quantification of Ca^2+^/ER fluorescence intensity ratios is shown in the bar graph (Right). F) Levels of IP3R and VDAC1 were detected by WB. G) Representative IF images showing co‐localization of IP3R (Red) and VDAC1 (Green) (Left). Scale bar, 10 µm. Manders' overlap coefficient is shown in the bar graph (Right). H) Representative IF images showing glucose uptake assessed using the 2‐NBDG probe (Green). Scale bar, 10 µm. I) Relative mRNA levels of glycolysis and oxidative phosphorylation genes were quantified by qRT‐PCR. *N* = 3. J) Enzymatic activities of HK2, PFKL, and PKM2 were measured. *N* = 3. K,L) 2‐DG (50 µM, 24 h) was used to inhibit glycolysis. K) Levels of p‐NLRP3^S295^ and inflammasome proteins were detected by WB. L) Representative TEM images showing MAM architecture in cells (Left). Quantification of ER–Mito distances is presented in the bar graph (Right). Scale bar, 2 µm (overview) and 1 µm (zoom). Data are presented as mean ± SD. *, *p* < 0.05, compared to the control or corresponding group.

The mitochondria–ER contact site (MAM) orchestrates inter‐organellar communication and governs xenobiotics exposure‐induced cell metabolism and regulatory cell death (RCD). GO and KEGG analyses of intersecting DEGs derived from MASLD mouse liver transcriptomics (GSE279512) and LPS‐induced human peripheral blood monocytes (GSE9916) revealed significant enrichment in “regulation of interleukin‐1 beta production”, “calcium‐mediated signaling”, “extracellular exosome”, “endoplasmic reticulum lumen”, and “NOD‐like receptor signaling pathway” (**Figure** **3**A; Figure S3A, Supporting Information), implicating MAM‐centered glycolytic reprogramming in iHD‐EVs biology. IF imaging showed pronounced recruitment of p‐NLRP3^S295^ to MAMs in iHD‐EVs‐treated THP‐1 macrophages versus cHD‐EV controls (Figure 3B; Figure S3B, Supporting Information). Concomitantly, iHD‐EVs elevated ER stress markers phosphorylated eIF2α (p‐eIF2α^S51^), IRE1α, and PERK (Figure 3C) and increased the p‐Drp1^S616^/p‐Drp1^S637^ ratio (Figure 3D), indicating compromised mitochondrial quality control (MQC) and disrupted MAM integrity.

We next interrogated MAM‐resident Ca^2+^ homeostasis. iHD‐EVs treatment triggered ER Ca^2+^ overload in THP‐1 cells (Figure 3E), paralleled by decreased IP3R and VDAC1 protein levels and reduced IP3R–VDAC1 co‐localization (Figure 3F,G; Figure S3C, Supporting Information). Since NLRP3 activation polarizes macrophages toward the pro‐inflammatory M1 phenotype and couples mitochondrial glycolysis to inflammasome output,^[^ ^31^, ^32^ ^]^ we examined hexokinase 2 (HK2), a key gatekeeper of the IP3R–VDAC1 complex.^[^ ^33^ ^]^ Live‐cell glucose uptake assays with fluorescent analog 2‐NBDG revealed increased glucose flux in iHD‐EV‐treated THP‐1 cells (Figure 3H). Compared with the cHD‐EV group, transcript levels of *GLUT1*, *HK2*, *PFKL*, *PKM2*, and *LDHA* were significantly up‐regulated in iHD‐EV‐treated THP‐1 cells (Figure 3I), paralleled by elevated ATP and lactic acid production and increased activities of glycolysis‐related enzymes (HK2, PFKL, and PKM2) (Figure S3D,E, Supporting Information; Figure 3J).

To establish causality of glycolysis, we blocked glycolysis with 2‐deoxy‐D‐glucose (2‐DG). 2‐DG blunted iHD‐EV‐induced p‐NLRP3^S295^ activation, reduced inflammasome proteins, and rescued normal mitochondria‐ER contact distances (Figure 3K,L). To assess the requirement of Ca^2+^ overload in p‐NLRP3^S295^‐dependent metaflammation, the cell‐permeable Ca^2+^ chelator BAPTA‐AM was used. Chelation of cytosolic Ca^2+^ with BAPTA‐AM attenuated iHD‐EV‐induced ER Ca^2+^ overload (Figure S3F, Supporting Information) and suppressed p‐NLRP3^S295^ signaling and inflammation in THP‐1 cells (Figure S3G–I, Supporting Information). Collectively, these results indicate that iHD‐EVs disrupt MAM integrity and function, trigger ER Ca^2+^ overload, and couple mitochondrial glycolytic reprogramming to p‐NLRP3^S295^‐dependent macrophage metaflammation.

### 2.4 PP2A‐B56δ Governs MAM Ca^2+^ Homeostasis in iHD‐EV‐Driven Macrophage Pyroptotic Metaflammation

**Figure 4:**
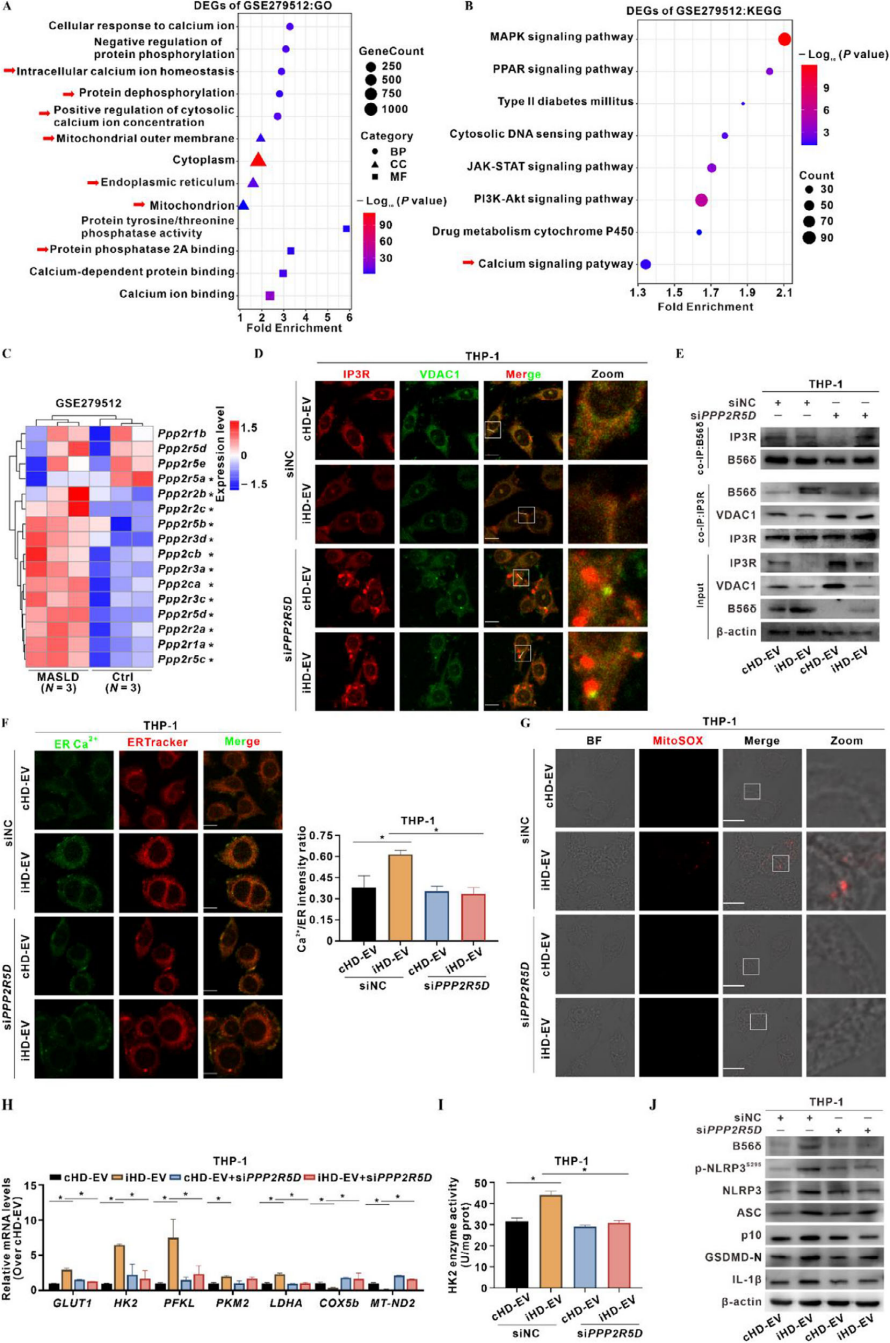
PP2A‐B56δ governs MAM Ca^2+^ homeostasis in iHD‐EV‐driven macrophage pyroptotic metaflammation. A–C) MASLD mouse liver transcriptomic data were retrieved from the GEO database (GSE279512; Ctrl, *N* = 3; MASLD, *N* = 3). GO A) and KEGG B) enrichment analyses were performed on DEGs. C) Heatmap of PP2A subunits expression is shown. D–J) THP‐1 cells were treated with cHD‐EV or iHD‐EV (40 µg mL^−1^, 4 h). B56δ was down‐regulated by si*PPP2R5D* (0.05 µM), with scrambled siNC (0.05 µM) as a negative control. D) Representative IF images showing co‐localization of IP3R (Red) and VDAC1 (Green) in cells. Scale bar, 10 µm. E) Input levels of IP3R, VDAC1, and B56δ proteins were detected by WB. Co‐immunoprecipitation (IP) shows IP3R‐associated B56δ and VDAC1‐associated B56δ/IP3R. F) Representative IF images showing the level of ER Ca^2+^ (Fluo‐5N AM, Green) (Left); ER are labeled with ERTracker (Red). Scale bar, 10 µm. Quantification of Ca^2+^/ER fluorescent intensity ratios is presented in the bar graph (Right). G) Representative bright‐field (BF) and IF images showing mitochondrial ROS (mtROS, MitoSOX Red). Scale bar, 10 µm. H) Relative mRNA levels of glycolysis and oxidative phosphorylation genes were quantified by qRT‐PCR. *N* = 3. I) Enzymatic activity of HK2 was measured. *N* = 3. J) Levels of B56δ, p‐NLRP3^S295^, and inflammasome proteins were detected by WB. Data are presented as mean ± SD. *, *p* < 0.05, compared to the control or corresponding group.

GO and KEGG interrogation of MASLD mouse livers (GSE279512) highlighted enrichment of “protein phosphatase 2A (PP2A) binding”, “negative regulation of protein phosphorylation”, and “calcium ion homeostasis” (**Figure** **4**A,B), implicating PP2A‐mediated dephosphorylation in MAM homeostasis and p‐NLRP3^S295^ activation. PP2A, a ubiquitously expressed serine/threonine phosphatase, orchestrates >90 % of cellular dephosphorylation events. Prior work links PP2A to IP3R–VDAC1 tethers at MAM,^[^ ^34^ ^]^ indicating that PP2A dysregulation could uncouple MAM Ca^2+^ flux from p‐NLRP3^S295^ activation. To identify the PP2A regulatory B‐subunit, we profiled B‐family transcripts during NLRP3 activation (Figure S4A, Supporting Information). *PPP2R5D* gene‐encoded PP2A‐B56δ (B56δ) was the most prominently up‐regulated subunit, a finding corroborated by GEO dataset (GSE279512) from MASLD mouse livers (Figure 4C). B56δ up‐regulation was NLRP3 activation‐dependent in both LPS+Nig‐treated model and iHD‐EV‐exposed THP‐1 cells (Figure S4B,C, Supporting Information). IF imaging revealed enriched B56δ localization at MAMs in iHD‐EV‐treated macrophages versus cHD‐EV controls (Figure S4D–F, Supporting Information), identifying B56δ as a candidate regulator for macrophage p‐NLRP3^S295^ signaling.

siRNA‐mediated B56δ knockdown (Figure S4G,H, Supporting Information) restored IP3R–VDAC1 proximity (Figure 4D,E; Figure S4I,J, Supporting Information), alleviated MAM Ca^2+^ overload (Figure 4F), and reduced mitochondrial ROS (mtROS) (Figure 4G). Concomitantly, B56δ silencing down‐regulated glycolytic genes and decreased HK2 enzymatic activity (Figure 4H,I). Mechanistically, B56δ depletion diminished p‐NLRP3^S295^ abundance at MAMs (Figure S4K–M, Supporting Information) and suppressed p‐NLRP3^S295^‐dependent inflammasome assembly and macrophage pyroptosis (Figure 4J; Figure S4N,O, Supporting Information). Collectively, these results indicate that iHD‐EVs induced B56δ up‐regulation and MAM translocation, thereby disrupting Ca^2+^ homeostasis, fueling mitochondrial glycolytic reprogramming, and licensing p‐NLRP3^S295^‐dependent pyroptotic metaflammation in macrophages.

### 2.5 iHD‐EVs Propagate Macrophage p‐NLRP3^S295^‐Dependent Metaflammation via an EV‐miR‐328‐3p/B56δ Axis

**Figure 5:**
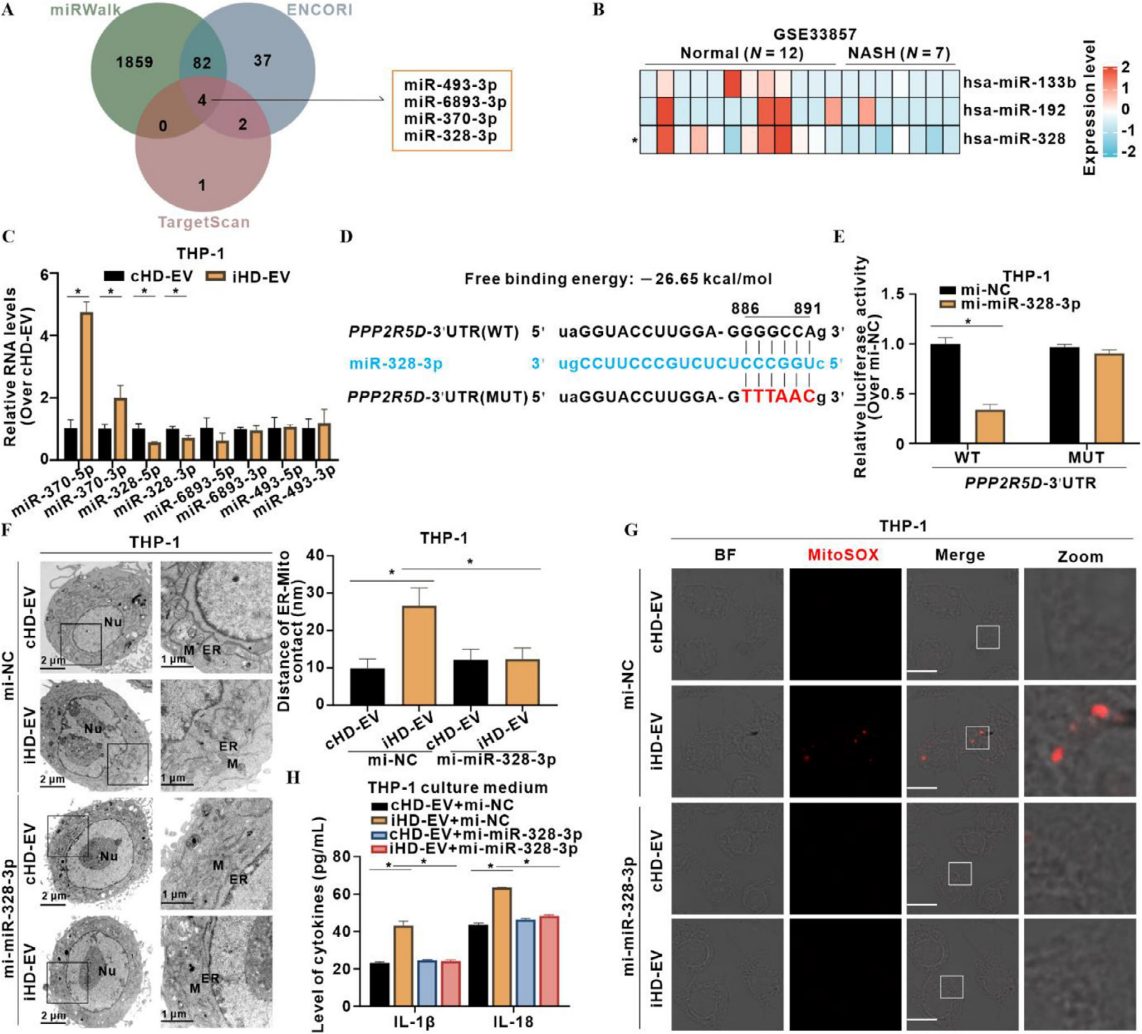
iHD‐EVs propagate macrophage p‐NLRP3^S295^‐dependent metaflammation via an EV‐miR‐328‐3p/B56δ axis. A) *PPP2R5D* 3′‐UTR‐targeting miRNAs were identified using miRWalk, ENCORI, and TargetScan databases. Four shared candidate miRNAs are displayed. B) Non‐coding RNA sequencing data from patients with NASH were retrieved from the GEO database (GSE33857; Normal, *N* = 12; NASH, *N* = 7). Relative expression of has‐miR‐133b, has‐miR‐192, and has‐miR‐328 is presented in a heatmap; candidate has‐miR‐6893, has‐miR‐493, and has‐miR‐370 were not detected in this dataset. C) THP‐1 cells were treated with iHD‐EV (40 µg mL^−1^, 4 h) to establish a metaflammatory macrophage model or with cHD‐EV (40 µg mL^−1^, 4 h) as control. Candidate miRNA levels were quantified by qRT‐PCR. *N* = 3. D) Predicted miR‐328‐3p binding site (WT) and seed‐sequence mutant (MUT) in *PPP2R5D* 3′‐UTR. Free binding energy was calculated with miRanda. E–H) THP‐1 cells were co‐transfected with *PPP2R5D* 3′‐UTR (WT or MUT) luciferase reporters and miR‐328‐3p mimic (mi‐miR‐328‐3p, 0.05 µM) for 24 h. E) Relative luciferase activity is shown in the bar graph. *N* = 3. F) Representative TEM images showing MAM architecture (Left). Scale bar, 2 µm (overview) and 1 µm (zoom). Quantification of ER–Mito distances is presented in the bar graph (Right). G) Representative bright‐field (BF) and IF images of mtROS (MitoSOX, Red). Scale bar, 10 µm. H) IL‐1β and IL‐18 levels in culture medium were quantified by ELISA. *N* = 3. Data are presented as mean ± SD. *, *p* < 0.05, compared to the control or corresponding group.

Emerging evidence positions EV‐encapsulated miRNAs as upstream transducers of xenobiotic‐induced liver injury. Indeed, the positive enrichment of the “pri‐miRNA transcription” pathway was apparent among the top GO terms in the DEGs of MASLD‐related macrophages (Figure 3A), prompting us to query whether iHD‐EV miRNAs serve as early indicators of MASLD. In silico integration of the miRWalk, ENCORI, and TargetScan databases identified four miRNAs—miR‐493‐5p, miR‐6893‐3p, miR‐370‐3p, and miR‐328‐3p—predicted to target the *PPP2R5D* 3′UTR (Figure 5A). Mining of the human EV transcriptome dataset (GSE33857) revealed that the miR‐328 cluster was markedly down‐regulated in EVs from patients with NASH, whereas the remaining candidates were undetectable (**Figure** **5**B). Consistently, the expression levels of the miR‐328 cluster, including miR‐328‐3p and miR‐328‐5p, were down‐regulated in iHD‐EV‐treated THP‐1 macrophages relative to cHD‐EVs, with miR‐328‐3p showing the most pronounced reduction; in contrast, the remaining candidate miRNAs (miR‐493, miR‐370, and miR‐6893 clusters) showed no significant difference (Figure 5C; Figure S5A, Supporting Information).

To verify the functional role of miR‐328‐3p, we engineered HepaRG cells to overexpress (with mimic, mi‐miR‐328‐3p) or silence (with inhibitor, in‐miR‐328‐3p) miR‐328‐3p and confirmed corresponding miR‐328‐3p levels in EVs derived from these cells (Figure S5B,C, Supporting Information). THP‐1 macrophages were co‐cultured with HepaRG cells overexpressing or silencing miR‐328‐3p. Low EV‐miR‐328‐3p enhanced, whereas high EV‐miR‐328‐3p suppressed, p‐NLRP3^S295^ activation and inflammasome proteins in co‐cultured THP‐1 macrophages (Figure S5D, Supporting Information). Similarly, direct transfection of THP‐1 cells with mi‐miR‐328‐3p reduced the *PPP2R5D* mRNA level (Figure S5E,F, Supporting Information), suppressed mitochondrial glycolysis reprogramming, and decreased HK2 enzymatic activity, leading to a decreased release of IL‐1β and IL‐18, whereas the in‐miR‐328‐3p exerted the opposite effect (Figure S5G–K, Supporting Information). Dual‐luciferase reporter assays confirmed direct binding of miR‐328‐3p to the 3′UTR of *PPP2R5D* mRNA with a predicted free energy of −26.65 kcal mol^−1^ (Figure 5D,E). miR‐328‐3p overexpression reduced iHD‐EV‐induced B56δ translocation to MAMs (Figure S5L, Supporting Information), preserved MAM architecture (Figure 5F), suppressed HK2 enzymatic activity (Figure S5M,N, Supporting Information), and reduced mitochondrial ROS (mtROS) generation (Figure 5G). Consequently, IL‐1β and IL‐18 release was significantly reduced upon high miR‐328‐3p expression (Figure 5H). Collectively, these results suggest that miR‐328‐3p is down‐regulated in iHD‐EVs, thereby derepressing B56δ translation, and promoting macrophage p‐NLRP3^S295^‐dependent metaflammation via the EV‐miR‐328‐3p/B56δ axis.

### 2.6 Targeting miR‐328‐3p or p‐NLRP3^S295^ Suppresses MC‐LR‐Induced Macrophage Metaflammation

**Figure 6:**
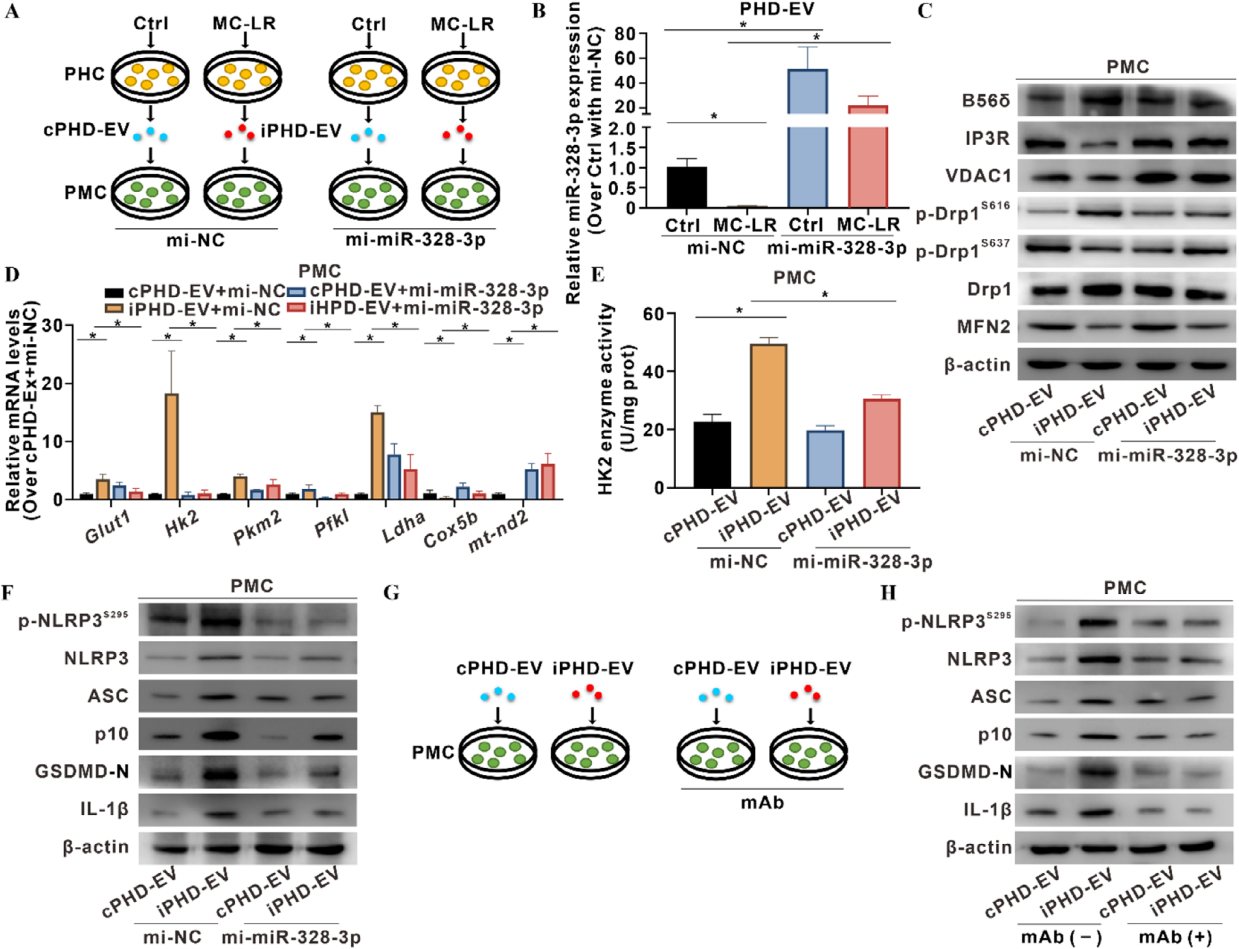
Targeting miR‐328‐3p or p‐NLRP3^S295^ suppresses MC‐LR‐induced macrophage metaflammation. Primary hepatocytes (PHCs) and hepatic primary macrophages (PMCs) were isolated from untreated C57BL/6J mice by sequential differential and Percoll density centrifugation. PHCs were exposed to MC‐LR (0.05 µM, 24 h) to generate inducible PHC‐derived EVs (iPHD‐EV); untreated PHCs provided control PHC‐derived EVs (cPHD‐EV) for PMCs treatment. A) Schematic diagram of *ex vivo* miR‐328‐3p intervention in PHD‐EV‐treated PMCs. B–F) PMCs were transfected with miR‐328‐3p mimic (mi‐miR‐328‐3p) or negative control mimic (mi‐NC). B) Relative miR‐328‐3p levels were quantified by qRT‐PCR. *N* = 3. C) Levels of B56δ, IP3R, VDAC1, and MQC‐related proteins were detected by WB. D) Relative mRNA levels of glycolysis and oxidative phosphorylation genes were quantified by qRT‐PCR. *N* = 3. E) Enzymatic activity of HK2 was measured. *N* = 3. F) Levels of p‐NLRP3^S295^ and inflammasome proteins were detected by WB. G,H) PMCs were treated with cPHD‐EV or iPHD‐EV. Anti‐p‐NLRP3^S295^ mAb conjugated to eTAT (100 µg mL^−1^, 24 h, referred to as mAb) was applied as an intervention. G) Schematic diagram of *ex vivo* mAb intervention in PHD‐EV‐treated PMCs. H) Levels of p‐NLRP3^S295^ and inflammasome proteins were detected by WB. Data are presented as mean ± SD. *, *p* < 0.05 compared to the control or corresponding group.

To verify the miR‐328‐3p/PP2A‐B56δ axis in regulating p‐NLRP3^S295^‐dependent metaflammation, we isolated murine primary hepatocytes (PHCs) and liver‐resident primary macrophages (PMCs) (**Figure** **6**A). Compared with control PHC‐derived EVs (cPHD‐EVs), MC‐LR‐induced PHC‐derived EVs (iPHD‐EVs) exhibited a significant decrease in the level of miR‐328‐3p, an effect that was reversed by transfection with mi‐miR‐328‐3p (Figure 6B). Concomitantly, mi‐miR‐328‐3p significantly down‐regulated *Ppp2r5d* mRNA levels in recipient PMCs (Figure S6A, Supporting Information). Mechanistically, overexpression of miR‐328‐3p rescued the IP3R–VDAC1 interaction (Figure S6B, Supporting Information), decreased mitochondrial fission (Figure 6C), and inhibited mitochondrial glycolytic metabolic reprogramming as well as HK2 activity in iPHD‐EV‐treated PMCs (Figure 6D,E). Furthermore, miR‐328‐3p overexpression decreased p‐NLRP3^S295^ activation and inflammasome protein levels (Figure 6F), reduced p‐NLRP3^S295^ localization to MAMs (Figure S6C,D, Supporting Information), and decreased GSDMD translocation to the plasma membrane in iPHD‐EV‐treated PMCs (Figure S6E,F, Supporting Information).

To specifically block macrophage p‐NLRP3^S295^‐dependent inflammation, we engineered a monoclonal antibody (anti‐p‐NLRP3^S295^ mAb) and delivered it into PMCs via an enhanced TAT‐based (eTAT) intracellular delivery system (designated the mAb group) (Figure 6G).^[^ ^35^ ^]^ Relative to iPHD‐EVs treatment alone, mAb administration markedly decreased p‐NLRP3^S295^ activation and inflammasome protein levels (Figure 6H), diminished p‐NLRP3^S295^ localization to MAMs (Figure S6G,H, Supporting Information), and reduced GSDMD translocation to the plasma membrane in iPHD‐EV‐treated PMCs (Figure S6I,J, Supporting Information). These results were corroborated in four additional models: (i) MC‐LR‐exposed co‐cultures subjected to mi‐miR‐328‐3p intervention (Figure S7A–I, Supporting Information); (ii) PP2A‐B56δ knockdown in PMCs (Figure S8A–G, Supporting Information); (iii) anti‐p‐NLRP3^S295^ mAb intervention in PMCs (Figure S9A–H, Supporting Information); and (iv) anti‐p‐NLRP3^S295^ mAb treatment of iHD‐EV‐challenged THP‐1 macrophages (Figure S10A–C, Supporting Information). Collectively, these data establish that the EV‐miR‐328‐3p/B56δ axis is both necessary and sufficient to license hepatic macrophage p‐NLRP3^S295^‐dependent metaflammation upon MC‐LR exposure, identifying a tractable therapeutic target for MASLD prevention and treatment.

### 2.7 In Vivo EV‐miR‐328‐3p/B56δ Axis Governs MC‐LR‐Induced p‐NLRP3^S295^‐Dependent Hepatic Metaflammation

**Figure 7:**
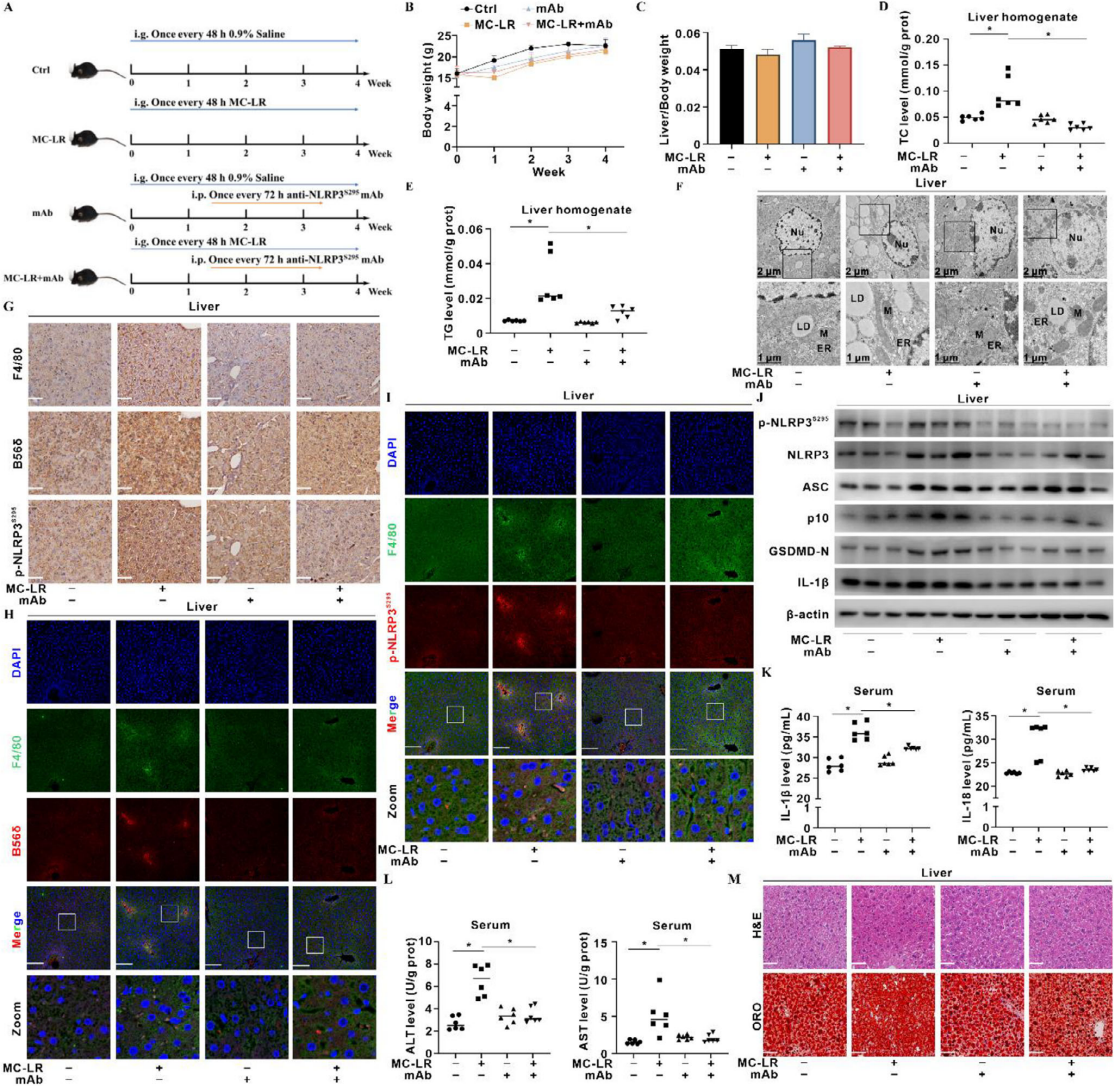
In vivo EV‐miR‐328‐3p/B56δ axis governs MC‐LR‐induced p‐NLRP3^S295^‐dependent hepatic metaflammation. Eight‐week‐old C57BL/6J mice were randomly divided into four groups. *N* = 6 per group. Mice in the MC‐LR group received MC‐LR (100 µg kg^−1^ body weight) by intragastric gavage every 48 h for 4 weeks to establish an exposure model; the control (Ctrl) group received an equal volume 0.9 % saline. After 2 weeks of MC‐LR exposure, the mAb‐intervention group received anti‐p‐NLRP3^S295^ mAb (10 mg kg^−1^ body weight) conjugated to eTAT by intraperitoneal injection every 72 h for 2 weeks. A) Schematic diagram of the in vivo exposure and intervention protocol. B,C) Average body‐weight B) and liver‐to‐body‐weight ratio C) were recorded. D,E) Hepatic TC D) and TG E) levels in liver homogenates were measured. F) Representative TEM images showing hepatic lipid droplets (LD). Scale bar, 2 µm (overview) and 1 µm (zoom). Nu, nucleus; ER, endoplasmic reticulum; M, mitochondria. G) Representative IHC staining images showing of F4/80, B56δ, and p‐NLRP3^S295^ expression and distribution. Scale bar, 50 µm. H,I) Representative IF images showing co‐localization of F4/80 (Green) with B56δ H) and p‐NLRP3^S295^ I) in liver sections; nuclei were counterstained with DAPI (Blue). Scale bar, 50 µm. J) Levels of p‐NLRP3^S295^ and inflammasome proteins in liver lysates were detected by WB. K) Serum IL‐1β (Left) and IL‐18 (Right) levels were quantified by ELISA. L) Serum ALT (Left) and AST (Right) activities were determined. M) Representative H&E and Oil Red O (ORO) staining images showing inflammatory foci and hepatic lipid accumulation in liver sections. Scale bar, 50 µm. Data are presented as mean ± SD or as scatter‐dot plots. *, *p* < 0.05 compared to the control or corresponding group.

To translate our mechanistic finding on targeted intervention against p‐NLRP3^S295^‐dependent metaflammation mediated by the EV‐miR‐328‐3p/B56δ axis into an in vivo setting, we conducted the animal experiment as outlined in **Figure** **7**A. Upon MC‐LR exposure, mice exhibited transient body‐weight loss; this decline was reversed by anti‐p‐NLRP3^S295^ mAb co‐administration (Figure 7B). Liver‐to‐body‐weight ratios remained comparable across groups (Figure 7C). MC‐LR exposure elevated hepatic TC and TG levels, whereas mAb restored these parameters to control values (Figure 7D,E), a finding corroborated by TEM imaging of reduced hepatic lipid droplets (Figure 7F). Hepatic EVs were isolated from control (Ctrl‐EVs) and MC‐LR‐exposed (MC‐LR‐EVs) mice and validated by TEM and nanoparticle‐tracking analysis (Figure S11A‐B, Supporting Information). Consistent with our in vitro data, the miR‐328 cluster was markedly decreased in MC‐LR‐EVs compared with Ctrl‐EVs (Figure S11C, Supporting Information). IHC staining showed that MC‐LR exposure increased the expression and distribution of F4/80, B56δ, and p‐NLRP3^S295^; these increases were abolished by mAb co‐treatment (Figure 7G). IF imaging further showed that mAb co‐treatment diminished the co‐localization of F4/80 with either B56δ or NLRP3 (Figure 7H,I). mAb administration attenuated the MC‐LR‐induced upregulation of p‐NLRP3^S295^ activation and inflammasome proteins (Figure 7J). Serum levels of IL‐1β, IL‐18, ALT, and AST were increased in the MC‐LR‐exposed group and were significantly ameliorated by mAb treatment (Figure 7K,L). Histological assessment showed fewer inflammatory foci and reduced lipid accumulation in mAb‐treated livers (Figure 7M). Collectively, these results identify p‐NLRP3^S295^ as a potential druggable target for mitigating MC‐LR‐induced MASLD.

To determine whether mAb exerts its protective effects via hepatic macrophages, we isolated primary mPMCs from the livers of each group. The levels of B56δ, IP3R, VDAC1, and MQC proteins in mPMCs mirrored out in vitro signatures (Figure S11D, Supporting Information). Concomitantly, p‐NLRP3^S295^ activation and inflammasome proteins were upregulated in MC‐LR‐exposed mPMCs and down‐regulated by mAb treatment (Figure S11E, Supporting Information). MC‐LR exposure triggered a glycolytic shift in mPMCs—evidenced by increased HK2 activity and elevated ATP and lactic acid production—resulting from decreased IP3R–VDAC1 interaction (Figure S11F–I, Supporting Information). MC‐LR exposure promoted p‐NLRP3^S295^ translocation to MAMs in mPMCs, an effect attenuated by mAb treatment (Figure S11J, Supporting Information). Collectively, these data indicate that mAb alleviates hepatic metaflammation by suppressing macrophage‐specific, p‐NLRP3^S295^‐dependent inflammasome regulating cascades.

Finally, to translate our mechanistic findings to the clinical setting, we initiated a prospective cohort study at Xiang'an Hospital of Xiamen University. After excluding secondary causes of hepatic steatosis and individuals with incomplete data, we enrolled twenty‐three adult patients undergoing clinically indicated hepatic resection who provided histologically confirmed liver tissue along with matched fresh serum samples. Serum MC‐LR concentrations were quantified by ultra‐high performance liquid chromatography–tandem mass spectrometry (uHPLC). Participants identified as MASLD were stratified into low‐exposure (<50 ng L^−1^, *N* = 3) and high‐exposure (≥50 ng L^−1^, *N* = 10) groups based on serum MC‐LR levels (Figure S12A, Supporting Information). EVs isolated from serum were used to measure EV‐miR‐328‐3p levels. qRT‐PCR revealed a 13.04‐fold reduction in EV‐miR‐328‐3p levels within serum EVs from the high MC‐LR group compared with the low exposure group (Figure S12B, Supporting Information). Additionally, immunohistochemistry of paired liver tissue samples showed increased in p‐NLRP3^S295^ staining in the high MC‐LR group (Figure S12C, Supporting Information). Pearson correlation analysis demonstrated a positive relationship between serum MC‐LR level and hepatic p‐NLRP3^S295^ expression (*r* = 0.7423, *p* < 0.05) (Figure S12D, Supporting Information). Moreover, we prospectively enrolled age‐ and sex‐matched control (*N* = 10) undergoing clinically indicated hepatic resection. Serum and liver tissue samples were collected for analysis. Histopathological assessment (H&E and Sirius red staining) confirmed MASLD hallmarks—steatosis, inflammatory foci, hepatocyte vacuolization, and pericellular fibrosis (Figure S12E, Supporting Information)—graded according to the SAF/FLIP algorithm (Table S1, Supporting Information).^[^ ^36^ ^]^ qRT‐PCR revealed a 4.31‐fold decrease in EV‐miR‐328‐3p levels in MASLD‐derived EVs compared with controls (Figure S12F, Supporting Information). Concomitantly, immunohistochemistry of paired liver tissue samples showed elevated hepatic p‐NLRP3^S295^ expression in MASLD samples (Figure S12G, Supporting Information). Pearson correlation analysis further disclosed a significant inverse correlation between EV‐miR‐328‐3p and hepatic p‐NLRP3^S295^ (*r* = –0.7346, *p* < 0.05) (Figure S12H, Supporting Information). These expanded human data corroborate our mechanistic findings and support the translational potential of serum EV‐miR‐328‐3p as a novel biomarker for p‐NLRP3^S295^‐dependent metaflammation in MASLD progression. Collectively, elevated MC‐LR exposure is associated with decreased serum EV‐miR‐328‐3p and heightened p‐NLRP3^S295^‐dependent metaflammation in MASLD progression, validating the clinical relevance of our mechanistic framework.

## 3 Discussion

MC‐LR is a prominent CEC that contributes to the development of systemic diseases. MASLD, a multifactorial disorder driven by inflammation, lipotoxicity, fibrosis, and other pathogenic cues, is positively associated with exposure to environmental hepatotoxicants such as MC‐LR. Beyond hepatitis and carcinogenesis, MC‐LR injures the kidneys, brain, and respiratory epithelium following aerosol exposure.^[^ ^37^, ^38^, ^39^ ^]^ These multi‐organ toxicities underscore the urgent need to elucidate MC‐LR pathogenesis and devise effective interventions. Previous studies have shown that even low‐dose MC‐LR provokes hepatocyte pyroptosis, activates the unfolded protein response, and upregulates lipogenic genes, thereby promoting lipid accumulation, fibrosis, and steatohepatitis.^[^ ^40^, ^41^ ^]^ Moreover, pre‐existing MASLD amplifies MC‐LR hepatotoxicity on a lipotoxic and inflammatory background, indicating a bidirectional, mutually promoting relationship between MC‐LR exposure and metabolic liver disease.^[^ ^42^ ^]^ The NLRP3 inflammasome drives MASLD progression, and its inhibition ameliorates hepatic inflammatory injury in mice.^[^ ^43^ ^]^ In this study, using in vitro and in vivo models of low‐dose MC‐LR exposure, we demonstrate that iHD‐EVs initiate macrophage p‐NLRP3^S295^‐dependent metaflammation. In co‐culture systems (HepaRG–THP‐1 and PHC–PMC), iHD‐EVs—but not EVs derived from lung, kidney, and neuronal cells—activated the miR‐328‐3p/PP2A‐B56δ/p‐NLRP3^S295^ axis in THP‐1 cells (data shown in response letter). Site‐specific dephosphorylation at S295 suppressed NLRP3 inflammasome activation in iHD‐EV‐treated macrophages. In MC‐LR‐exposed mice, hepatic inflammatory injury was paralleled by elevated p‐NLRP3^S295^ expression and distribution, whereas therapeutic administration of anti‐p‐NLRP3^S295^ mAb markedly alleviated MC‐LR‐induced hepatic inflammation. Collectively, chronic low‐dose MC‐LR accumulation accelerates MASLD progression through iHD‐EV‐mediated hepatocyte–macrophage communication, highlighting iHD‐EVs as critical drivers of p‐NLRP3^S295^‐dependent inflammation and identifying a new avenue for preventing MC‐LR‐induced hepatotoxicity.

Macrophages orchestrate the inflammatory microenvironment and initiate damage repair during MASLD progression. Hepatocyte‐derived EVs transport hepatokines—including proteins, lipids, and RNAs—to modulate hepatic metabolism and inflammatory responses. Alterations in EV abundance or cargo disrupt inter‐cellular and inter‐organ communication in metabolic diseases.^[^ ^44^, ^45^ ^]^ For example, hepatic lipid accumulation drives a pro‐inflammatory macrophage cascade through miR‐192‐5p released from lipotoxic hepatocytes, highlighting the central role of hepatocyte‐derived EVs in liver inflammation beyond classical cytokine signaling. miR‐328‐3p has been implicated in regulating malignant phenotypes and glycolysis in cancer cells.^[^ ^46^ ^]^ Circular RNA_0 02178 is reported to compete with miR‐328‐3p to promote tumor growth and inflammation,^[^ ^47^ ^]^ and the lncRNA PFI/miR‐328‐3p/AGO‐2 axis regulates MLE‐12 cell apoptosis during lung injury.^[^ ^48^ ^]^ Here, we identify miR‐328‐3p as a novel regulator of macrophage p‐NLRP3^S295^‐dependent metaflammation. Downregulated miR‐328‐3p in hepatocyte‐derived EVs enhances glucose flux, upregulates macrophage glycolytic rate‐limiting enzymes to rewire glycolysis, and drives p‐NLRP3^S295^‐dependent inflammation, positioning miR‐328‐3p as a candidate circulating biomarker of hepatic inflammation in MASLD. Although previous studies show that miRNAs can directly target *NLRP3* mRNA,^[^ ^49^, ^50^ ^]^ we uncover an indirect mechanism: hepatocyte‐derived miR‐328‐3p binds the 3′‐UTR of PP2A‐B56δ mRNA. Its down‐regulation elevates PP2A‐B56δ expression, which dephosphorylates IP3R and disrupts MAM Ca^2+^ homeostasis. Ca^2+^ overload at MAMs subsequently activates macrophage p‐NLRP3^S295^‐dependent metaflammation. These findings establish MAMs as a metaflammatory signaling platform in hepatocyte–macrophage communication and highlight miRNA‐mediated regulation at this interface as a driver of MASLD progression.

Maintenance of cellular architecture and function relies on intimate inter‐organellar communication; ER‐mitochondria crosstalk in now recognized as a central driver of metabolic diseases. Although MC‐LR induces hepatotoxicity by promoting mitochondrial fission, the precise mechanism by which MC‐LR disrupts organelle crosstalk has not been fully elucidated.^[^ ^51^ ^]^ MAMs—the physical tethers between ER and mitochondria—govern inflammatory signaling, cholesterol transport, and Ca^2+^ flux. Pharmacologic or genetic interventions targeting mitochondria fission (e.g., Drp1^S616^ phosphorylation) or Ca^2+^ release (e.g., NLRP3 inflammasome blockade) attenuates pyroptosis,^[^ ^52^, ^53^ ^]^ indicating MAM Ca^2+^ dyshomeostasis as a proximal trigger for NLRP3 activation. Intracellular K^+^ and Ca^2+^ fluctuations modulate NLRP3 activation, and disruption of IP3R–VDAC1‐mediated Ca^2+^ transport suppresses ASC oligomerization and caspase‐1 processing.^[^ ^54^ ^]^ NLRP3 activation requires a priming signal and a second activation cue, both of which are governed by PTMs.^[^ ^55^ ^]^ Using PHC–PMC co‐cultures, we confirmed that the EV‐miR‐328‐3p/PP2A‐B56δ axis fine‐tunes p‐NLRP3^S295^ signaling (Figures S7 and S8, Supporting Information). Earlier work showed that diacylglycerol recruits Golgi apparatus‐localized PKD to phosphorylate NLRP3^S295^ under stress.^[^ ^56^ ^]^ In this study, BAPTA‐AM‐mediated Ca^2+^ chelation validate that Ca^2+^ flux is indispensable for p‐NLRP3^S295^‐dependent metaflammation. MC‐LR is a potent inhibitor of protein phosphatases PP1 and PP2A. PP2A dynamically dephosphorylates NLRP3^S5^ within the pyrin domain, reducing charge‐charge repulsion and facilitating NLRP3‐ASC oligomerization and activation.^[^ ^26^, ^57^ ^]^ During priming, PP2A counteracts the inhibitory phosphorylation imposed by TBK1 and IKKε; during assembly, PP2A displace IKKα from the ASC‐containing pre‐complex to enable full inflammasome formation.^[^ ^58^ ^]^ Recent evidence further indicates that TRPV1‐mediated Ca^2+^ influx promotes PP2A phosphorylation and activation in microglia, highlighting a Ca^2+^–PP2A axis in neuro‐inflammation.^[^ ^59^ ^]^ Importantly, PP2A specificity is dictated by its B‐subunits. We previously demonstrated that PP2A‐B55δ dephosphorylates COX‐2 to license NLRP3 activation in aflatoxin B1 (AFB1)‐induced liver inflammatory injury.^[^ ^60^ ^]^ By contrast, PP2A‐B56δ suppresses fatty acid oxidation and promote glycolytic reprogramming in injured tubular cells by dephosphorylating ACC and GLUT1, thereby preventing Trim21‐mediated ubiquitination and proteasomal degradation of GLUT1 during acute or chronic kidney injury.^[^ ^61^ ^]^ Similarly, oxidative phosphorylation blockade triggers a PP2A‐B56δ‐dependent death pathway in tumor cells through ROS‐mediated dissociation of CIP2A from PP2A, suggesting extensive crosstalk between PP2A‐B56δ and glycolysis.^[^ ^62^ ^]^ In the present study, we uncover an additional layer of PP2A‐B56δ regulation: the miR‐328‐3p/PP2A‐B56δ axis disrupts IP3R–VDAC1 tethering at MAMs, provoking Ca^2+^ overload and glycolytic metabolic reprogramming that licenses p‐NLRP3^S295^ recruitment and activation. While our current data focus on phosphorylation and metabolic control, the established PP2A‐B56δ‐mediated suppression of ubiquitin‐dependent GLUT1 degradation^[^ ^61^ ^]^ and the emerging role of autophagy in restraining NLRP3 inflammasome activity prompt us to speculate that PP2A‐B56δ may also modulate NLRP3 stability through ubiquitination or selective autophagy. Future work will employ ubiquitin‐remnant profiling and autophagic flux assays to explicitly test these possibilities. Collectively, low‐dose MC‐LR exposure upregulates macrophage PP2A‐B56δ expression via hepatocyte‐EVs delivery of miR‐328‐3p, thereby disrupting the IP3R–VDAC1 complex, inducing MAM Ca^2+^ overload, and driving p‐NLRP3^S295^‐dependent metaflammation. These findings highlight the dose‐dependent complexity of xenobiotic toxicity and establish the miR‐328‐2p/PP2A‐B56δ axis as a novel targetable checkpoint for preserving MAM homeostasis and restraining macrophage inflammation in MASLD.

Current MASLD therapy remains limited to lifestyle adjustments; pharmacological options are scarce and frequently confounded by adverse events. Growing evidence positions metabolic‐inflammatory crosstalk as a pivotal node for drug discovery. NLRP3‐null mice display improved insulin sensitivity, underscoring the physiological role of NLRP3 in metabolic homeostasis.^[^ ^63^ ^]^ The small‐molecule NLRP3 inhibitor CY‐09, which occupies the NACHT domain and blocks ATPase activity, attenuates hepatic steatosis in experimental MASLD mice.^[^ ^64^, ^65^ ^]^ Reciprocally, the balance between glycolysis and oxidative phosphorylation dictates macrophage polarization; enhanced glycolysis promotes macrophage NLRP3 activation and M1 polarization, exacerbating hepatic inflammatory damage.^[^ ^66^ ^]^ In contrast, M2 macrophage‐EVs carrying miR‐690 improve mouse glucose tolerance and insulin sensitivity through the NADK pathway, whereas EV‐loaded hydrogels modulate metabolic homeostasis and restrain pyroptosis, highlighting engineered miRNA‐delivery systems as a promising therapeutic avenue for inflammatory liver diseases.^[^ ^67^, ^68^ ^]^ Here, we leveraged MAM as a metabolic sensing platform and functional coupled NLRP3 activation to ER Ca^2+^ overload signaling. The miR‐328‐3p/PP2A‐B56δ axis propagated a pro‐inflammatory microenvironment by driving macrophage glycolysis and inflammasome activation. To circumvent off‐target effects of inherent to miRNA therapy, we directly targeted p‐NLRP3^S295^, a MAM‐localized signature of Ca^2+^ overload. An anti‐p‐NLRP3^S295^ mAb was efficiently internalized into macrophages or administered into mice via an eTAT delivery system, blocked p‐NLRP3^S295^‐dependent inflammasome activation, and curtailed pro‐inflammatory cytokine release, demonstrating that site‐specific neutralization of p‐NLRP3^S295^ alleviates xenobiotic‐driven chronic metaflammation. These proof‐of‐concept data provide a rationale for developing novel liver‐targeted, site‐specific anti‐p‐NLRP3^S295^ mAbs for MASLD prevention and treatment. Preliminary toxicological assessment of this mAb has been conducted, and the recombinant anti‐p‐NLRP3^S295^ mAb effectively ameliorated HFD‐induced hepatic inflammation and lipid accumulation‐related injury.^[^ ^35^ ^]^ Humanized antibody‐drug conjugates are currently under development to enhance precision and clinical translatability for MASLD therapy.

## 4 Conclusion 

In summary, we demonstrate that the miR‐328‐3p/PP2A‐B56δ axis disrupts MAM integrity in macrophages and triggers p‐NLRP3^S295^‐dependent metaflammation. Chronic, low‐dose MC‐LR exposure aggravates hepatic inflammatory injury via hepatocyte‐derived EVs. Reduced EV‐miR‐328‐3p levels serve as a circulating biomarker of macrophage p‐NLRP3^S295^‐dependent metaflammation, whereas site‐specific anti‐p‐NLRP3^S295^ mAbs effectively ameliorate metaflammatory damage in experimental MASLD. Our findings underscore the urgent need to mitigate environmental exposures that accelerate MASLD progression and position the miR‐328‐3p/PP2A‐B56δ/p‐NLRP3^S295^ signaling cascade as a tractable therapeutic target for preventing and treating environmentally driven metabolic liver disease.

## 5 Experimental Section

### Bioinformatics Analyses

Publicly available mRNA expression datasets (GSE9916 and GSE279512) were retrieved from the GEO database of NCBI (http://www.ncbi.nlm.nih.gov/geo/). Differentially expressed genes (DEGs) were defined using a threshold of |log_2_FC| ≥1.5 and an adjusted *p*‐value <0.05. Raw data were normalized and expression values calculated with the limma package in R software (v4.2.0). Gene Ontology (GO) and KEGG pathway analyses were performed and visualized using the ggplot2 R package.^[^ ^69^ ^]^ Putative miRNAs targeting the 3′UTR of *PPP2R5D* mRNA were predicted using ENCORI, miRWalk, and TargetScan. The free binding energy between the *PPP2R5D* 3′UTR and miR‐328‐3p was estimated using miRanda (http://www.microRNA.org).

### Human Serum and Liver Specimens

Serum and liver tissue samples (control, *N* = 10. MASLD, *N* = 13) were collected form patients undergoing clinically indicated hepatic resection at Xiang'an hospital of Xiamen university. Written informed consent was obtained from all participants. The protocol was approved by the Medical Ethics Committee of Xiamen University School of Medicine (Approval No. XAHLL20233004, 1 August 2023). Serum MC‐LR was quantified by ultra‐high performance liquid chromatography–tandem mass spectrometry (uHPLC‐MS/MS). Extracellular vesicles (EVs) were isolated from serum and miR‐328‐3p levels were quantified by qRT‐PCR. Liver specimens were processed for hematoxylin and eosin (H&E) and Sirius red staining; histological features were graded according to the SAF/FLIP algorithm (Table S1, Supporting Information).^[^ ^36^ ^]^ p‐NLRP3^S295^ expression was assessed by immunohistochemistry (IHC) staining.

### Mouse models of Metaflammation and Treatments

Eight‐week‐old C57BL/6J mice (SLAC Laboratory Animal Co., Ltd., Shanghai, China) were randomly assigned to four groups (*N* = 6 per group). The MC‐LR group received 100 µg kg^−1^ body‐weight MC‐LR (in 0.9 % saline) by oral gavage every 48 h for 4 weeks to mimic dietary/water‐borne exposure.^[^ ^70^ ^]^ An anti‐p‐NLRP3^S295^ monoclonal antibody (mAb, generated in‐house) was conjugated to an enhanced TAT‐based (eTAT) intracellular delivery system prior to injection (mAb group).^[^ ^35^ ^]^ After 2 weeks of MC‐LR exposure, mice in the intervention group (MC‐LR + mAb) received 100 µg kg^−1^ mAb (i.p.) every 72 h for 2 weeks. All mice were euthanized 24 h after the final dose. IHC and immunofluorescence (IF) assays were used to map indicated protein expression. Liver sections were stained with H&E and Oil Red O (ORO). Serum obtained by retro‐orbital bleeding was used to quantify biochemical markers and inflammatory cytokines. Total liver lysates were prepared for Western blotting (WB). All procedures were approved by the Experimental Animal Ethics Committee of Xiamen University (Approval No. XMULAC20220282, March 12 2022).

### Cell Culture

Human THP‐1 monocytes and engineered human embryonic kidney HEK‐293T cells were maintained in RPMI 1640 (Gibco, Thermo Fisher, Waltham, MA, USA) supplemented with 10 % (v/v) fetal bovine serum and 1 % penicillin–streptomycin. THP‐1 cells were differentiated into M0 macrophages by 24‐h exposure to 100 ng mL^−1^ PMA (CSNpharm, Chicago, IL, USA). All cell lines were cultured at 37 °C in a humidified 5 % CO_2_ incubator (Thermo Fisher). Primary hepatocytes (PHCs) and hepatic primary macrophages (PMCs) were isolated as previously described.^[^ ^60^ ^]^ Briefly, PHCs and PMCs were separated by sequential low‐speed centrifugation and Percoll density‐gradient centrifugation (Cytiva, Marlborough, MA, USA). Cells were then seeded on culture dishes pre‐coated with collagen type I (dissolved in 0.1 % glacial acetic acid) for subsequent experiments. All reagents and antibodies used in this study are listed in Table S2 (Supporting Information).

### Plasmid Construction and siRNA Transfection

Plasmids pCDH‐CD63‐RFP, pB513B‐NLRP3‐GFP, pCR3.1‐NLRP3^S295>A^ (site‐directed mutation of S295 to alanine), and pCR3.1‐NLRP3^S295>D^ (site‐directed mutation of S295 to aspartate) were constructed using the KOD Plus Mutagenesis Kit (Toyobo, Osaka, Japan) and were maintained in‐house. miR‐328‐3p mimic (mi‐miR‐328‐3p), inhibitor (in‐miR‐328‐3p), and their corresponding negative controls (mi‐NC, in‐NC), as well as *PPP2R5D*‐targeting siRNA (si*PPP2R5D*) and non‐targeting control siRNA (siNC), were purchased from RiboBio (Guangzhou, Guangdong, China). The *PPP2R5D* 3′‐UTR was amplified from HEK‐293T genomic DNA, digested with corresponding restriction enzymes (New England Biolabs, Ipswich, MA, USA), and ligated into pmirGLO using T4 DNA ligase (New England Biolabs) to generate pmirGLO‐*PPP2R5D*‐3′UTR (WT). The mutant reporter construct, pmirGLO‐*PPP2R5D*‐3′UTR (MUT), was generated by inversed PCR following the manufacturer's protocol (YeaSen Biotech, Shanghai, China). Cells were transfected with plasmids or siRNA using Lipofectamine 2000 (Thermo Fisher) according to the manufacturer's instructions. All primers used in this study were synthesized by Sangon Biotech (Shanghai, China) and are listed in Table S3 (Supporting Information).

### Isolation and Characterization of Hepatocyte‐Derived EVs (HD‐EVs)

HepaRG cells were exposed to MC‐LR (0.05 µM, for 24 h) to establish the exposure model. Conditioned medium was collected and subjected to sequential ultracentrifugation (OPTIMA L‐90K, Beckman Coulter, Brea, CA, USA) to isolate EVs, as previously described.^[^ ^71^, ^72^ ^]^ EV identity was confirmed by: transmission electron microscope (TEM) for cup‐shaped morphology, nanoparticle tracking analysis (Nano‐Z90, Malvern Panalytical, Malvern, UK) for size distribution, and WB for canonical markers (CD9, CD81, TSG101, and CD63).

### Live‐Cell Imaging of NLRP3 Inflammasome‐Speck Formation

THP‐1 cells were seeded on glass‐bottom dishes and transfected with pB513B‐NLRP3‐GFP. Time‐lapse images of NLRP3 inflammasome‐speck formation were captured with a Zeiss Celldiscoverer 7 fully automated live‐cell imaging system (Carl Zeiss AG, Oberkochen, Germany) under the indicated treatments.

### Dual‐Luciferase Reporter Assay

The dual‐luciferase reporter assay was performed with the Dual‐Luciferase Reporter Assay System (Beyotime, Shanghai, China) according to the manufacturer's protocol. Briefly, THP‐1 cells were co‐transfected with either pmirGLO‐*PPP2R5D*‐3′UTR (WT) or pmirGLO‐*PPP2R5D*‐3′UTR (MUT) together with mi‐NC or mi‐miR‐328‐3p. Firefly and Renilla luciferase activities were measured on a GloMax® 96 microplate luminometer (Promega, Madison, WI, USA) and normalized to Renilla activity.

### Western Blot (WB) Analysis

Procedures were performed as previously described.^[^ ^73^ ^]^ Briefly, proteins were separated by SDS‐PAGE and transferred to PVDF membranes (0.45 µm, Merck‐Millipore). Membranes were blocked and incubated overnight at 4 °C with the indicated primary antibodies (e.g., anti‐p‐NLRP3^S295^, ‐NLRP3, and ‐ASC, etc.). After washes with TBST, membranes were incubated with HRP‐conjugated corresponding secondary antibodies and visualized with a chemiluminescence imaging system (Azure c‐300; Azure Biosystems, Dublin, CA, USA).

### Co‐Immunoprecipitation (co‐IP) Assays

THP‐1 cells were lysed in ice‐cold NP‐40 lysis buffer and clarified by centrifugation. SureBeads Protein A/G magnetic beads (MedChemExpress, Monmouth Junction, NJ, USA) were pre‐incubated with the indicated antibodies (anti‐phospho‐Ser/Thr, anti‐B56δ, or anti‐IP3R) for 10 min at room temperature, followed by incubation with clarified lysate for 1 h at 4 °C on a rotating platform. Beads were washed with PBST (0.1 % Tween‐20 in PBS) and eluted in 1× SDS loading buffer at 70 °C for 10 min. Eluates were analyzed by WB.

### Immunofluorescence (IF) Assays

THP‐1 cells were mounted on coverslips in 24‐well plates and treated as indicated. After washing with ice‐cold PBS, cells were fixed with pre‐chilled methanol at −20 °C for 20 min, blocked with 1 % (w/v) bovine serum albumin (BSA) in PBS for 30 min, and incubated overnight at 4 °C with the indicated primary antibodies (1:400 dilution), including anti‐NLRP3 (for inflammasome foci and MAM distribution), anti‐GSDMD (for co‐localization with Dio membrane dye), anti‐B56δ (for MAM translocation), and anti‐IP3R and anti‐VDAC1 (for IP3R–VDAC1 complex co‐localization). After washing, cells were incubated with the corresponding fluorescent secondary antibodies for 1 h at room temperature in the dark. Nuclei were counterstained with DAPI for 5 min. Ca^2+^ and mitochondrial ROS (mtROS) were labeled with probes according to the manufacturer's instructions. Images were captured on a Zeiss LSM 880^+^ Airyscan confocal microscope (Carl Zeiss AG, Oberkochen, Germany) and analyzed with ZEN 2.0 software.

### Immunohistochemistry (IHC) Staining

Mouse liver specimens were fixed in 4 % (w/v) paraformaldehyde overnight, dehydrated, and paraffin‐embedded for sectioning. IHC staining was conducted on the sections as previously described.^[^ ^69^ ^]^ Sections were incubated overnight at 4 °C with the indicated antibodies (anti‐F4/80, ‐B56δ, or ‐p‐NLRP3^S295^). Immune complexes were detected with HRP‐conjugated secondary antibodies and visualized with DAB. Slides were imaged on a Nikon DS‐Fi2 microscope (Nikon, Tokyo, Japan).

### Enzyme‐Linked Immunosorbent Assay (ELISA)

Levels of IL‐1β and IL‐18 in THP‐1 conditioned medium and mouse serum were quantified with commercial ELISA kits (ABclonal, Wuhan, Hubei, China) according to the manufacturer's instructions.

### Statistical Analysis

All raw datasets were pre‐processed for outlier detection and normalization. Experiments were repeated independently at least twice with a minimum of three biological replicates per group. All quantitative data are presented as mean ± standard deviation (SD). Two‐group comparisons were performed with unpaired two‐tailed Student's *t*‐tests (α = 0.05). Multiple‐group comparisons were performed by one‐way analysis of variance (ANOVA) followed by Tukey's post‐hoc (α = 0.05). Analyses and graphs were generated with GraphPad Prism 8.0 (GraphPad Software, San Diego, CA, USA). Statistical significance was set at *p* < 0.05 (*).

## Conflict of Interest

The authors declare no conflict of interest.

## Author Contributions

J.‐S.W., X.‐Y.Z., and X.‐Y.M. contributed equally to this paper. J.‐S.W. performed conceptualization, investigation, and wrote original draft; X.‐Y.Z. performed investigation, formal analysis; X.‐Y.M. performed investigation, formal analysis; Y.‐Y.W. performed investigation, formal analysis; L.‐L.W. performed investigation, formal analysis; Z.‐B.D. performed formal analysis; X.‐G.X. performed methodology; L.C. performed methodology; D.‐B.G. performed methodology; H.‐Y.Z. performed formal analysis; Y.‐L.Y. performed project administration; W.‐G.L. acquired resources; Y.‐C.L. performed conceptualization, formal analysis, and Wrote, reviewed, and edited the draft; Z.‐N.L. performed conceptualization, acquired resources, wrote, reviewed, and edited the draft, performed visualization, project administration, and supervision.

## Compliance with Ethics Requirements

All animal procedures were approved by the Experimental Animal Ethics Committee of Xiamen University (Approval No. XMULAC20220282, 12 March 2022). The study protocol for human serum and liver specimens was approved by the Medical Ethics Committee of Xiamen University School of Medicine (Approval No. XAHLL20233004, 1 August 2023).

## Supporting information



Supporting Information

## Data Availability

The data that support the findings of this study are available from the corresponding author upon reasonable request.
