# Supplementary material for: Hepatocyte‐Derived Extracellular Vesicles Deliver miR‐328‐3p to Trigger PP2A‐B56δ–Mediated p‐NLRP3S295‐Dependent Metaflammation in Macrophages upon Microcystin‐LR Exposure
Source: Adv Sci (Weinh). 2025 Nov 19;13(6):e07039. doi: 10.1002/advs.202507039 (PMC12866802; doi:10.1002/advs.202507039)
Supplement: Supplementary file 1 — Supporting Information [file ADVS-13-e07039-s001.docx]

**Supplementary materials**

**Hepatocyte Extracellular Vesicles Deliver miR-328-3p to Induce PP2A-B56δ–Mediated p-NLRP3^S295^-Dependent Metaflammation in Macrophages During Microcystin-LR Exposure**

Jia-Shen Wu ^a, 1^, Xin-Yu Zhang ^a, 1^, Xin-Yu Ma ^a, 1^, Yue-Yue Wei ^a^, Lei-Lei Wang ^a^, Ze-Bang Du ^a^, Xiao-Gang Xia ^b^, Lin Che ^a, c^, Dong-Bei Guo ^a^, Han-Ying Zheng ^a^, You-Liang Yao ^a^, Wen-Gang Li ^b, *^, Yu-Chun Lin ^a, *^, Zhong-Ning Lin ^a, *^

^a^ State Key Laboratory of Vaccines for Infectious Diseases, Xiang An Biomedicine Laboratory, National Innovation Platform for Industry-Education Integration in Vaccine Research, School of Public Health, Xiamen University, Xiamen, 361102, China.

^b^ Department of Hepatobiliary Surgery, Cancer Research Center, Xiang'an Hospital of Xiamen University, School of Medicine, Xiamen University, Xiamen, Fujian 361102, China.

^c^ State Key Laboratory of Oncology in South China, Guangdong Provincial Clinical Research Center for Cancer, Sun Yat-sen University Cancer Center, Guangzhou, 510060, China.

^1^ These authors contributed equally to this paper.

^*^ Corresponding Author: lwgang@xmu.edu.cn (W.-G. Li); Yu-Chun Lin, linych@xmu.edu.cn (Y.-C. Lin); Zhong-Ning Lin, linzhn@xmu.edu.cn (Z.-N. Lin).

Tel: +86 592 2880615; Fax: +86 592 2881578.

**1. Supplementary experimental section**

*1.1 Reagents and antibodies*: MC-LR was purchased from Cayman Chemicals (Ann Arbor, MI, USA). Palmitic acid (PA) was purchased from Sigma Aldrich (Saint Louis, MO, USA). Lipopolysaccharide (LPS), nigericin (Nig), 3, 3'-dioctadecyloxacarbocyanine perchlorates (Dio), 2-deoxy-D-glucose (2-DG), CY-09, GW4869, 1,2-bis (2-aminophenoxy) ethane-*N*, *N*, *N*′, *N*′-tetraacetic acid-acetoxymethyl ester (BAPTA-AM), and the 2-NBDG probe were purchased from MedChemExpress (Monmouth Junction, NJ, USA). The Dual-Luciferase Reporter Assay System and NP-40 lysis buffer were purchased from Beyotime (Shanghai, China). Percoll was purchased from Cytiva (Marlborough, MA, USA). The KOD plus Mutagenesis Kit was purchased from Toyobo (Osaka, JP). The DAB staining kit was purchased from MXB (Fuzhou, Fujian, China). The enhanced TAT-based intracellular delivery system (eTAT) was kindly provided by Prof. Sheng-Xiang Ge (Xiamen University, Fujian, China). ERTracker® Red and MitoSOX were purchased from Invitrogen (Carlsbad, CA, USA). MitoTracker® Green CMXRos was purchased from Cell Signaling Technology (Boston, MA, USA). Fluo-5N AM was purchased from Thermo Fisher Scientific (Waltham, MA, USA) for Ca^2+^ staining. The monoclonal antibody against p-NLRP3^S295^ (mAb@p-NLRP3^S295^) was prepared and validated as previously described.^[S1]^ HRP-conjugated goat anti-mouse and goat anti-rabbit IgG secondary antibodies, and Lipofectamine^TM^ 2000, were purchased from Thermo Fisher Scientific. Detailed information on the primary antibodies is provided in Table S2.

*1.2 Transmission electron microscopy (TEM)*: Mouse livers or THP-1 cells were fixed with 0.1 mol/L glutaraldehyde at 4 °C for 2 h and processed as previously reported.^[S2]^ EVs (20 μg/mL) were prepared by negative staining. Ultrastructure was examined using a Hitachi HT-7800 transmission electron microscope (HITACHI, Tokyo, JP).

*1.3 Cell viability assay*: HepaRG cells were treated with MC-LR at the indicated doses (0-31.25 μM, 24 h) or 0.05 μM MC-LR for the indicated times (0-24 h). Cell viability was tested using the MTS assay (Solarbio, Beijing, China) according to the manufacturer's instructions.

*1.4 Quantitative real-time reverse transcription PCR (qRT-PCR)*: Total RNA and EVs RNA were extracted using TRIzol reagent (TaKaRa, Shiga, JP) according to the manufacturer's protocol. cDNA was synthesized using the Evo M-MLV RT Premix (Accurate Biology, Changsha, Hunan, China); miRNA was reverse-transcribed with specific primers. qRT-PCR was performed on a Q-towere^3^G system (Analytik Jena, Jena, Germany) using SYBR Green SuperMix (Bio-Rad, Hercules, CA, USA). Gene expression was normalized to *ACTB*, *Gapdh*, or *U6*. Primer sequences are listed in Table S3.

*1.5 Biochemical analyses*: ALT and AST activities were measured using commercial kits (Nanjing JianCheng, Nanjing, China) according to the manufacturer's protocols. HK2, PFKL, and PKM2 enzymatic activities in THP-1 cells were determined using corresponding kits (Boxbio, Beijing, China). ATP and lactic acid levels were assessed using an ATP assay kit (Beyotime) and lactic acid assay kit (Nanjing JianCheng), respectively. Signals were read on a CLARIOstar microplate reader (BMG Labtech, Ortenberg, Germany).

*1.6 Oil Red O* (ORO) *staining*: Hepatic lipid droplets (LDs) were visualized with an ORO staining kit (Nanjing JianCheng) according to the manufacturer's protocol. Images were captured using a Nikon DS-Fi2 microscope (Nikon, Tokyo, JP).

**2. Supplementary tables**

**Table S1. Histopathological MASLD activity score (SAF/FLIP) of human liver samples from clinically indicated resections**

| ID | Steatosis | Inflammatory foci | Hepatic  vacuolization | Perisinusoidal  fibrosis | MASLD activity score | Diagnostic classification |
| --- | --- | --- | --- | --- | --- | --- |
| 1 | 0 | 0 | 0 | 0 | 0 | N |
| 2 | 0 | 0 | 0 | 0 | 0 | N |
| 3 | 0 | 0 | 0 | 0 | 0 | N |
| 4 | 0 | 0 | 0 | 0 | 0 | N |
| 5 | 0 | 0 | 0 | 0 | 0 | N |
| 6 | 0 | 0 | 0 | 0 | 0 | N |
| 7 | 0 | 0 | 0 | 0 | 0 | N |
| 8 | 0 | 0 | 0 | 0 | 0 | N |
| 9 | 0 | 0 | 0 | 0 | 0 | N |
| 10 | 0 | 0 | 0 | 0 | 0 | N |
| 11 | 2 | 1 | 2 | 1 | 6 | MASLD |
| 12 | 3 | 1 | 3 | 1 | 8 | MASLD |
| 13 | 1 | 1 | 1 | 2 | 5 | MASLD |
| 14 | 1 | 3 | 1 | 3 | 8 | MASLD |
| 15 | 3 | 3 | 3 | 1 | 10 | MASLD |
| 16 | 3 | 2 | 3 | 1 | 9 | MASLD |
| 17 | 1 | 1 | 1 | 3 | 6 | MASLD |
| 18 | 1 | 1 | 3 | 3 | 8 | MASLD |
| 19 | 1 | 1 | 2 | 3 | 7 | MASLD |
| 20 | 2 | 1 | 1 | 3 | 7 | MASLD |
| 21 | 2 | 1 | 1 | 3 | 7 | MASLD |
| 22 | 2 | 1 | 3 | 3 | 9 | MASLD |
| 23 | 2 | 1 | 2 | 2 | 7 | MASLD |

SAF/FLIP: **Steatosis, Activity, and Fibrosis/Fatty Liver Inhibition of Progression.** N: non-MASLD

**Table S2. Details of primary antibodies used in the present study**

| Primary Antibodies | Manufacturers | Code Number | Dilution/Concentration | | | Molecule weight | Species |
| --- | --- | --- | --- | --- | --- | --- | --- |
|  |  |  | WB | IF | IHC |  |  |
| AIM2 | Beyotime | AF6168 | 1:1000 |  |  | 47 kDa | Rabbit |
| ASC | Santa Cruz | sc-514414 | 1:1000 |  |  | 24 kDa | Mouse |
| B55β | ABclonal | A10494 | 1:1000 |  |  | 52 kDa | Rabbit |
| B56δ | ABclonal | A21122 | 1:1000 | 1:400 | 1:100 | 66 kDa | Rabbit |
| Caspase-1 | Santa Cruz | sc-56036 | 1:1000 |  |  | 45 kDa | Mouse |
| Caspase-3 | Beyotime | AC030 | 1:10000 |  |  | 35 kDa | Rabbit |
| CD206 | Abcam | ab300621 | 1:1000 |  |  | 165 kDa | Mouse |
| CD63 | Abcam | ab134045 | 1:1000 | 1:400 |  | 30 kDa | Rabbit |
| COX IV | Proteintech | 11242-1-AP | 1:1000 |  |  | 19.6 kDa | Rabbit |
| eIF2α | Cell Signaling | 9722 | 1:1000 |  |  | 38kDa | Rabbit |
| GSDMD | Proteintech | 20770-1-AP | 1:1000 | 1:400 |  | 53 kDa | Rabbit |
| IL-1β | Proteintech | 16806-1-AP | 1:1000 |  |  | 31 kDa | Rabbit |
| iNOS | Abcam | ab178945 | 1:1000 |  |  | 131 kDa | Rabbit |
| IP3R | Santa Cruz | sc-271197 | 1:1000 | 1:400 |  | 304 kDa | Mouse |
| IRE1α | ABclonal | A21021 | 1:1000 |  |  | 130 kDa | Rabbit |
| NLRP3 | Abclonal | A24294 | 1:1000 | 1:400 |  | 110 kDa | Mouse |
| NOD1 | ABclonal | A1246 | 1:1000 |  |  | 108 kDa | Rabbit |
| p-eIF2α^S51^ | Cell Signaling | 3398 | 1:1000 |  |  | 38 kDa | Mouse |
| PERK | ABclonal | A18196 | 1:1000 |  |  | 140 kDa | Rabbit |
| p-NLRP3^S295^ | Affinity Biosciences | AF4320 | 1:1000 | 1:400 | 1:100 | 118 kDa | Rabbit |
| TSG101 | BD Biosciences | 612696 | 1:1000 |  |  | 46 kDa | Mouse |
| VDAC1 | Santa Cruz | sc-390996 | 1:1000 | 1:400 |  | 35 kDa | Rabbit |
| F4/80 | Abcam | ab6640 |  | 1:400 |  | 117 kDa | Mouse |
| β-actin | Proteintech | 66009-1-Ig | 1:5000 |  |  | 42 kDa | Mouse |

| **Table S3. Primer sequences used in the present study** | |
| --- | --- |
| **Primer** | **Sequence (5'-3')** |
| *NLRP3*-S295-RP | gaagaggattctggagggttttctc |
| *NLRP3*-S295D-FP | GActcttcctcatggacggcttcgat |
| *NLRP3*-S295A-FP | GCctcttcctcatggacggcttcgat |
| WT-FP | GCTAGCTAGCCCCCTCACGTTCCTACCACA |
| WT-RP | ACGCGTCGACTTCTCAGAGACCCACTTTATTCAGTTC |
| MUT-FP | TACCTTGGAGTTTAACGGGGCTGAGGAAGGCC |
| MUT-RP | AGCCCCGTTAAACTCCAAGGTACCTATAAGGGACAAG |
| *ACTB* FP | CACCATTGGCAATGAGCGGTTC |
| *ACTB* RP | AGGTCTTTGCGGATGTCCACGT |
| *GLUT1* FP | GTGGGCATGTGCTTCCAGTAT |
| *GLUT1* RP | CAGCTCCTCGGGTGTCTTGT |
| *HK2* FP | TTCTTGGCCTTGGACCTTG |
| *HK2* RP | CCAGATGCCTTGAAGCCTTTT |
| *PFKL* FP | GCTGGGCGGCACTATCATT |
| *PFKL* RP | TCAGGTGCGAGTAGGTCCG |
| *LDHA* FP | CCAAGCTGGTCATTATCACGG |
| *LDHA* RP | CATTCCACTCCATACAGGCAC |
| *PKM2* FP | AAGGGTGTGAACCTTCCTGG |
| *PKM2* RP | GCTCGACCCCAAACTTCAGA |
| *COX5B* FP | TGTGAAGAGGACAATACCAGCG |
| *COX5B* RP | CCAGCTTGTAATGGGCTCCAC |
| *MT-ND2* FP | TCCGGCCTGCTTCTTCTCAC |
| *MT-ND2* RP | TTGCGTAGCTGGGTTTGGTTT |
| *PPP2R2A* FP | GAGAACAAAATCCAGTCTCATAG |
| *PPP2R2A* RP | GGCCTAAAGACTGGCACTCG |
| *PPP2R3A* FP | GGAGAAAGTTGCTGAATAACC |
| *PPP2R3A* RP | CTGAATAACCGTGGTG |
| *PPP2R4* FP | TCTCAGGCATACGCTGACTAC |
| *PPP2R4* RP | GGAGACTCTGTACTCGAAGGT |
| *PPP2R5A* FP | TGCTAACATCTTCCGTACACTTC |
| *PPP2R5A* RP | CCTCAAGCGTGGGTTCATCC |
| *PPP2R5B* FP | GCCCGTCTACCCAGACATC |
| *PPP2R5B* RP | TCAAGATTGGGCTCATCCTCT |
| *PPP2R5C* FP | CAAAGCCAATCCCCAGTAC |
| *PPP2R5C* RP | TCGGATCTTTCTGTGCCTGA |
| *PPP2R5D* FP | CCTTCATCGAATCCCACAGGG |
| *PPP2R5D* RP | TGTTTGGCTGGAAATCAGGAG |
| *PRKD1* FP | GTGAAGGGTGTGGTCTG |
| *PRKD1* RP | CTCTGATGGTGATTTTTGCTC |
| *U6* FP | CTCGCTTCGGCAGCACA |
| *U6* RP | AACGCTTCACGAATTTGCGT |
| miR-328-3p FP | ACACTCCAGCTGGGCTGGCCCTCTCTGC |
| miR-328-3p RP | CTCAACTGGTGTCGTGGAGTCGGCAATTCAGTTGAGACGGAAGG |
| miR-328-5p FP | ACACTCCAGCTGGGGGGGGGGCAGGAGGGGC |
| miR-328-5p RP | CTCAACTGGTGTCGTGGAGTCGGCAATTCAGTTGAGCCCTGAGC |
| miR-493-3p FP | ACACTCCAGCTGGGTGAAGGTCTACTGTGT |
| miR-493-3p RP | CTCAACTGGTGTCGTGGAGTCGGCAATTCAGTTGAGCCTGGCAC |
| miR-493-5p FP | ACACTCCAGCTGGGTTGTACATGGTAGGCT |
| miR-493-5p RP | CTCAACTGGTGTCGTGGAGTCGGCAATTCAGTTGAGAATGAAAG |
| miR-370-3p FP | ACACTCCAGCTGGGGCCTGCTGGGGTGGAA |
| miR-370-3p RP | CTCAACTGGTGTCGTGGAGTCGGCAATTCAGTTGAGACCAGGTT |
| miR-370-5p FP | ACACTCCAGCTGGGCAGGTCACGTCTCTGC |
| miR-370-5p RP | CTCAACTGGTGTCGTGGAGTCGGCAATTCAGTTGAGGTAACTGC |
| miR-6893-3p FP | ACACTCCAGCTGGGCCCTGCTGCCTTCACC |
| miR-6893-3p RP | CTCAACTGGTGTCGTGGAGTCGGCAATTCAGTTGAGCTGGCAGG |
| miR-6893-5p FP | ACACTCCAGCTGGGCAGGCAGGTGTAGGG |
| miR-6893-5p RP | CTCAACTGGTGTCGTGGAGTCGGCAATTCAGTTGAGGCTCCACC |
| *Gapdh* FP | CCCTTAAGAGGGATGCTGCC |
| *Gapdh* RP | ATGAAGGGGTCGTTGATGGC |
| *Ppp2r5d* FP | AAGAAGGATAAGCAGGAACCCTC |
| *Ppp2r5d* RP | CTGGGTCGCTTGTTGGATGA |
| *Glut1* FP | GCAGTTCGGCTATAACACTGG |
| *Glut1* RP | GCGGTGGTTCCATGTTTGATTG |
| *Hk2* FP | ATGATCGCCTGCTTATTCACG |
| *Hk2* RP | CGCCTAGAAATCTCCAGAAGGG |
| *Pfkl* FP | ACGTGAAGGATCTGGTGGTTC |
| *Pfkl* RP | GGTAGCCTCACAGACTGGTT |
| *Pkm2* FP | GTCCGCTCTAGGTATCGCAG |
| *Pkm2* RP | AAATGATGCCAGTGTTGCGG |
| *Ldha* FP | CAAAGACTACTGTGTAACTGCGA |
| *Ldha* RP | TGGACTGTACTTGACAATGTTGG |
| *Cox5b* FP | ATCTTGCTCAGCCTGTTCCCG |
| *Cox5b* RP | TTGTATGGGTCCAGTCCCTTCT |
| *Mt-nd2* FP | CAAGGGATCCCACTGCACATAG |
| *Mt-nd2* RP | AAGTCCTCCTCATGCCCCTATGA |

**Note:** All miRNA primers used in this study are conserved between human and mouse, except for the miR-6893 cluster, for which no mouse ortholog exists.

**3. Supplementary figures and legends**


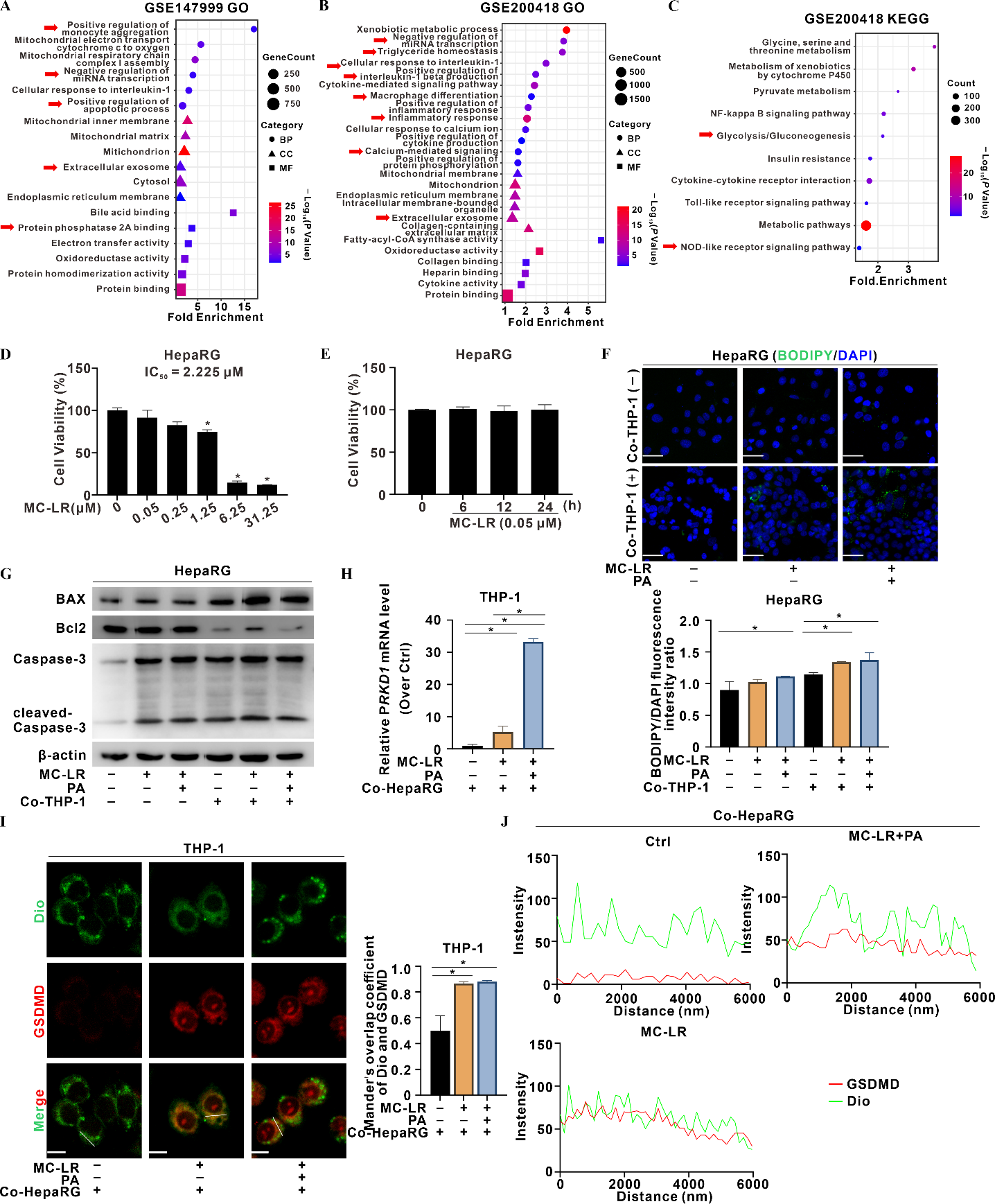


**Figure S1. MC-LR-exposed hepatocytes induce macrophage p-NLRP3^S295^-dependent inflammation. (A)** Dataset GSE147999 was derived from RNA sequencing profiles of HepaRG cells exposed to 10 μM MC-LR for 2 h (*N* = 19) or solvent controls (*N* = 36). **Differentially expressed genes (DEGs; |log₂FC| ≥ 1, FDR < 0.05) were subjected to** GO enrichment analysis to identify MC-LR-responsive pathways. **(B-C)** Dataset GSE200418 was derived from human precision-cut liver slices cultured for 48 h in lipotoxic medium (36 mM glucose, 5 mM fructose, 1 nM insulin, 480 μM oleic acid, and 240 μM palmitic acid) as the MASLD group (*N* = 6) or in 25 mM glucose alone as the control group (*N* = 6). GO (B) and KEGG (C) enrichment analyses of DEGs reveal pathways relevant to human MASLD pathogenesis. **(D)** HepaRG cells were exposed to graded doses of MC-LR (0.05-31.25 μM) for 24 h. Cell viability was measured by MTS assay. *N* = 3. **(E)** HepaRG cells were treated with 0.05 μM MC-LR for different time (6-24 h). Cell viability was measured by MTS assay. *N* = 3. **(F-I)** A Transwell co-culture system was established with HepaRG and THP-1 cells. HepaRG cells were treated for 24 h with DMSO as the control (Ctrl) group, MC-LR (0.05 μM) as the exposure group, or combined with MC-LR and PA (200 μM) as the MASLD group. **(F)** Representative IF images showing BODIPY-labeled lipid droplets (Green) in co-cultured HepaRG cells (Upper); nuclei were counterstained with DAPI (Blue). Scale bar, 20 μm. Quantification of the BODIPY/DAPI fluorescence intensity ratios is presented in the bar graph (Lower). **(G)** Levels of apoptosis-related proteins in co-cultured HepaRG cells were detected by WB. **(H)** Relative mRNA levels of *PRKD1* gene were quantified by qRT-PCR. *N* = 3. **(I-J)** Representative IF images showing co-localization of GSDMD (Red) with Dio-stained membranes (Green) (I, Left). Scale bar, 10 μm. Manders' overlap coefficients are presented in the bar graph (I, Right). **(J)** Immunofluorescence emission-spectrum profiles of GSDMD (Red) and Dio-stained membranes (Green) co-localization are shown. Data are presented as mean ± SD. *, *P* < 0.05, compared to the control or corresponding group.


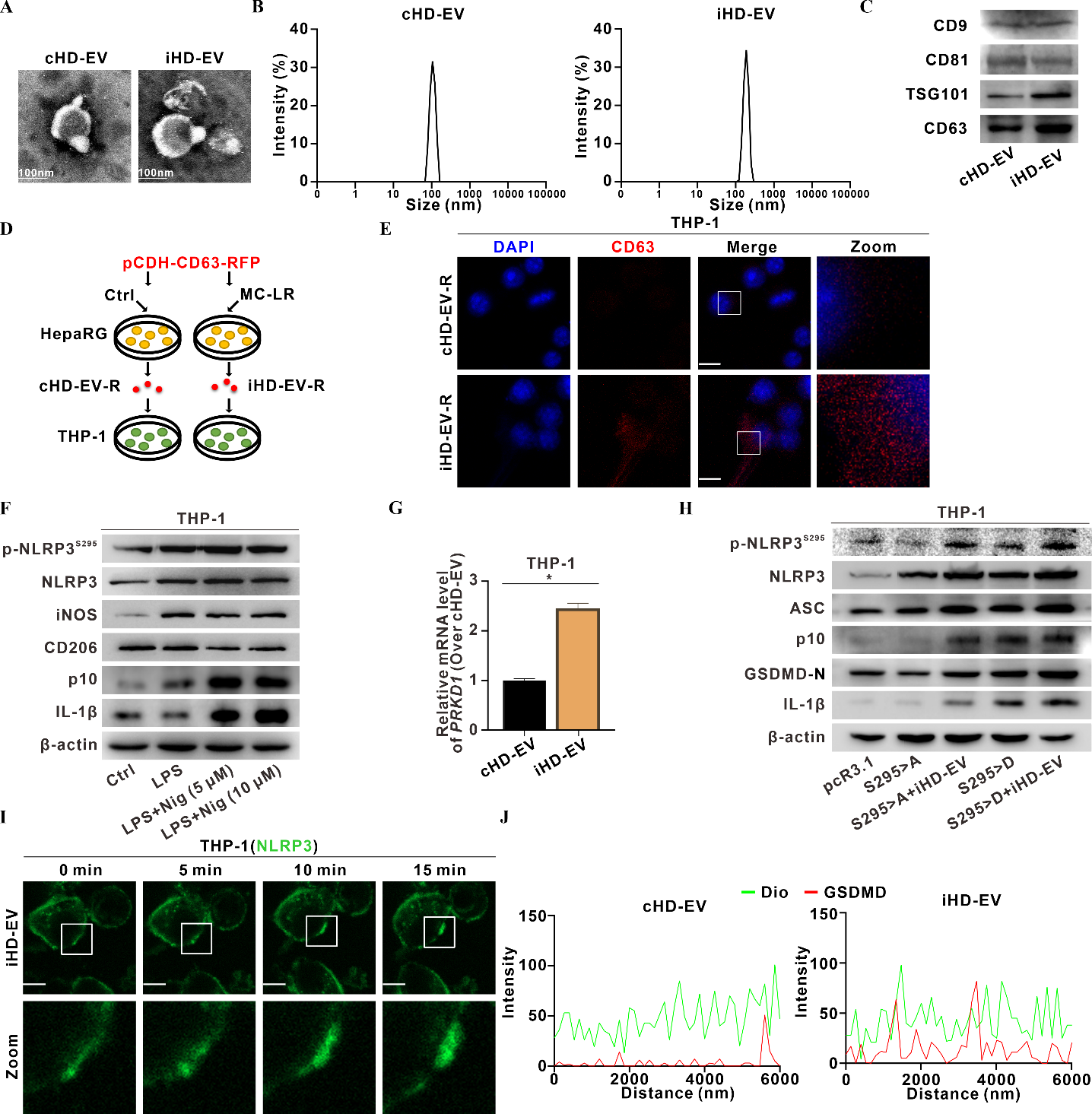


**Figure S2. MC-LR-induced iHD-EVs trigger macrophage p-NLRP3^S295^-dependent inflammation. (A)** Representative TEM images showing the morphology of cHD-EV (Left) and iHD-EV (Right). Scale bar, 100 nm. **(B)** Nanoparticle tracking analysis of size distributions for cHD-EV (Left) and iHD-EV (Right). **(C)** Levels of CD9, CD81, TSG101, and CD63 markers in the isolated EVs were detected by WB. **(D)** Schematic diagram of the uptake assay. HepaRG cells were transfected with pCDH-CD63-RFP and then treated with MC-LR (0.05 μM) for 24 h, and HD-EVs labeled with red fluorescence protein (RFP)-CD63 (cHD-EV-R and iHD-EV-R) were isolated for THP-1 uptake. **(E)** Representative IF images showing uptake of CD63-RFP-labeled HD-EVs; nuclei were counterstained with DAPI (Blue). Scale bar, 10 μm. **(F)** THP-1 cells were treated with LPS (100 ng/mL, 3 h) plus nigericin (Nig, 5-10 μM, 4h) to establish NLRP3 activation model. Levels of macrophage polarization markers (iNOS, CD206), p-NLRP3^S295^, and inflammasome proteins were detected by WB. **(G)** Relative levels of *PRKD1* mRNA were quantified by qRT-PCR. *N* = 3. **(H)** THP-1 cells were pre-transfected with pCR3.1-NLRP3^S295>A^ (S295>A) or pCR3.1-NLRP3^S295>D^ (S295>D) to mimic hypo- or hyper-phosphorylation at serine 295, respectively, with empty pcR3.1 as a negative control. THP-1 cells then treated with cHD-EV or iHD-EV (40 μg/mL, 4 h). Levels of p-NLRP3^S295^ and inflammasome proteins were detected by WB. **(I)** THP-1 cells were pre-transfected with pB513B-NLRP3 (Green) and followed by the treatment with iHD-EV (40 μg/mL). Representative live-cell time-lapse images showing NLRP3 inflammasome speck formation (5**–**15 min intervals) in THP-1 cells. Scale bar, 5 μm. **(J)** Immunofluorescence emission-spectrum profiles of GSDMD (Red) and Dio-stained membranes (Green) corresponding to Figure 2C. Data are presented as mean ± SD. *, *P* < 0.05, compared to the control or corresponding group.


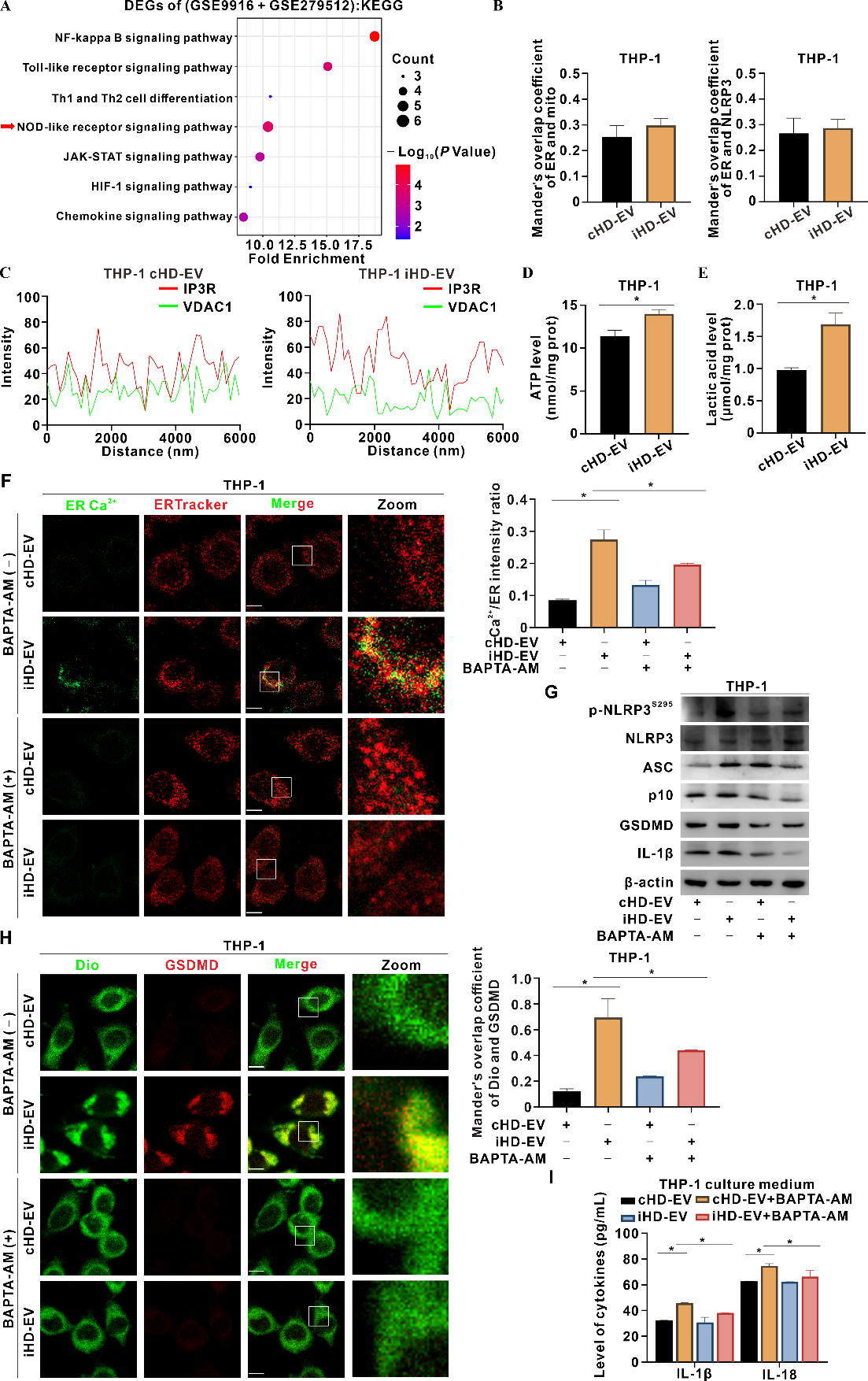


**Figure S3. iHD-EVs trigger MAM Ca^2+^ overload to activate macrophage p-NLRP3^S295^-dependent metaflammation. (A)** Data retrieved from GEO databases, based on LPS-activated human monocyte-macrophages (GSE9916) and MASLD mouse liver transcriptomics (GSE279512), were mined for shared DEGs. KEGG analysis identified enriched signaling pathways among the shared DEGs. **(B)** Manders' overlap coefficients corresponding to Figure 3B are presented in bar graphs. **(C)** Immunofluorescence emission-spectrum profiles of IP3R (Red) and VDAC1 (Green) co-localization corresponding to Figure 3G. **(D-E)** Levels of ATP (D) and lactic acid (E) were detected. *N* = 3. **(F-I)** BAPTA-AM (5 μM, 2 h) was used to chelate Ca^2+^. **(F)** Representative IF images showing the level of ER Ca^2+^ (Fluo-5N AM, Green) (Left); ER are labeled with ERTracker (Red). Scale bar, 10 μm. Quantification of Ca^2+^/ER fluorescent intensity ratios is presented in the bar graph (Right). **(G)** Levels of p-NLRP3^S295^ and inflammasome proteins were detected by WB. **(H)** Representative IF images showing GSDMD (Red) co-localization with Dio-labeled plasma membrane (Green) (Left). Scale bar, 10 μm. Manders' overlap coefficients are presented in the bar graph (Right). **(I)** IL-1β and IL-18 levels in culture medium were quantified by ELISA. *N* =3. Data are presented as mean ± SD. *, *P* < 0.05, compared to the control or corresponding group.


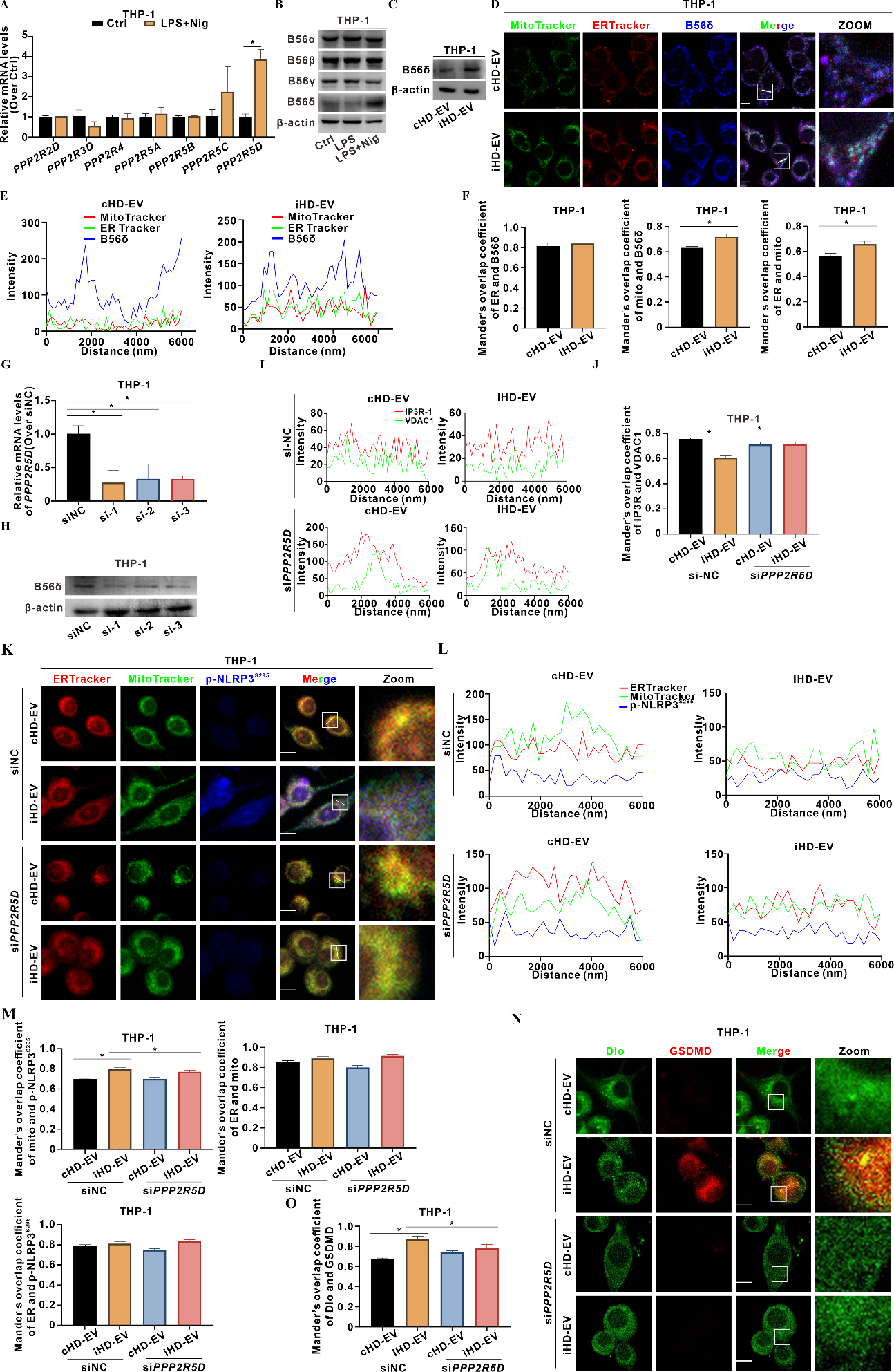


**Figure S4. PP2A-B56δ governs MAM Ca^2+^ homeostasis in iHD-EV-driven macrophage pyroptotic metaflammation. (A-B)** THP-1 cells were treated with LPS (100 ng/mL, 3 h) plus nigericin (Nig, 5-10 μM, 4h) to establish NLRP3 activation model. **(A)** Relative mRNA levels of different PP2A B-subunits were quantified by qRT-PCR. *N* = 3. **(B)** Levels of PP2A-B56 subunits were detected by WB. **(C)** Levels of B56δ in cHD-EV- or iHD-EV-treated THP-1 cells were detected by WB. **(D-F)** Representative IF images showing MAM localization of B56δ (Blue); mitochondria and ER are labeled with MitoTracker (Green) and ERTracker (Red) (D). Scale bar, 10 μm. Immunofluorescence emission spectra (E) and Manders' overlap coefficients (F) for PP2A-B56δ/MAM co-localization are shown. **(G-H)** THP-1 cells were transfected with si*PPP2R5D* (si-1, si-2, or si-3, 0.05 μM, 24 h) to knockdown B56δ, with scrambled siNC (0.05 μM, 24 h) as a negative control. Silence and knockdown efficiency were verified by qRT-PCR (G) and WB (H). **(I-J)** Immunofluorescence emission spectra (I) and Manders' overlap coefficients (J) for IP3R (Red)/VDAC1 (Green) co-localization corresponding to Figure 4D. **(K-M)** Representative IF images showing MAM localization of p-NLRP3^S295^ (Blue); mitochondria and ER are labeled with MitoTracker (Green) and ERTracker (Red) (K). Scale bar, 10 μm. Immunofluorescence emission spectra (L) and Manders' overlap coefficients (M) for p-NLRP3^S295^/MAM co-localization are shown. **(N-O)** Representative IF images showing GSDMD (Red) co-localization with Dio-stained membranes (Green) (N). Scale bar, 10 μm. Manders' overlap coefficients are presented in the bar graph (O). Data are presented as mean ± SD. *, *P* < 0.05, compared to the control or corresponding group.


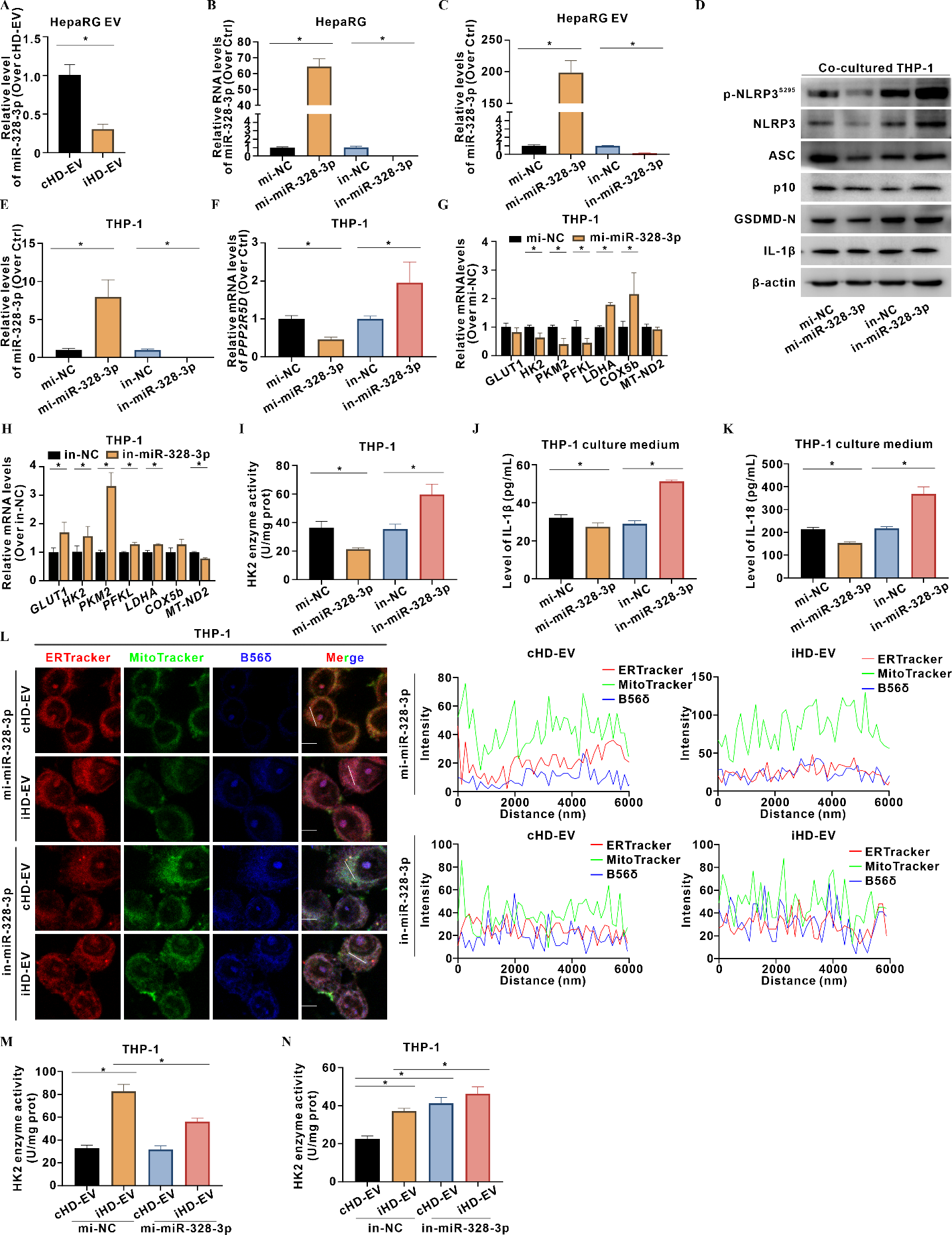


**Figure S5. miR-328-3p targets B56δ to regulate macrophage MAM homeostasis and p-NLRP3^S295^-dependent pyroptosis. (A)** Relative miR-328-3p levels in EVs derived from control or MC-LR-exposed (0.05 μM, 24 h) HepaRG cells (HepaRG EV) were quantified by qRT-PCR. *N* = 3. **(B-C)** HepaRG cells were transfected with miR-328-3p mimic (mi-miR-328-3p) or inhibitor (in-miR-328-3p) to generate high- and low-miR-328-3p expression models, using mi-NC or in-NC as controls. Relative miR-328-3p levels in HepaRG cells (B) and their HepaRG EVs (C) were quantified by qRT-PCR. *N* = 3. **(D)** THP-1 macrophages were co-cultured with high- or low-miR-328-3p expression HepaRG cells. Levels of p-NLRP3^S295^ and inflammasome proteins in co-cultured THP-1 cells were detected by WB. **(E-K)** THP-1 cells were transfected with mi-miR-328-3p (0.05 μM) or in-miR-328-3p (0.1 μM) for 24 h to generate high- or low-miR-328-3p expression macrophages, using mi-NC or in-NC as controls. **(E)** Relative miR-328-3p levels (E) and *PPP2R5D* mRNA levels (F) in THP-1 cells were quantified by qRT-PCR. *N* = 3. **(G-H)** Relative mRNA levels of glycolysis and oxidative phosphorylation genes in THP-1 cells were quantified by qRT-PCR. *N* = 3. **(I)** HK2 enzymatic activity was measured. *N* = 3. **(J-K)** IL-1β and IL-18 levels in culture medium were quantified by ELISA. *N* = 3. **(L-N)** THP-1 cells with high- or low-miR-328-3p expression were treated with cHD-EVs or iHD-EVs. **(L)** Representative IF images showing MAM localization of B56δ (Blue); mitochondria and ER are labeled with MitoTracker (Green) and ERTracker (Red), respectively (Left). Scale bar, 10 μm. Immunofluorescence emission-spectrum profiles for B56δ/MAM co-localization are presented in bar graphs (Right). **(M-N)** HK2 enzymatic activity was measured. *N* = 3. Data are presented as mean ± SD. *, *P* < 0.05, compared to the control or corresponding group.


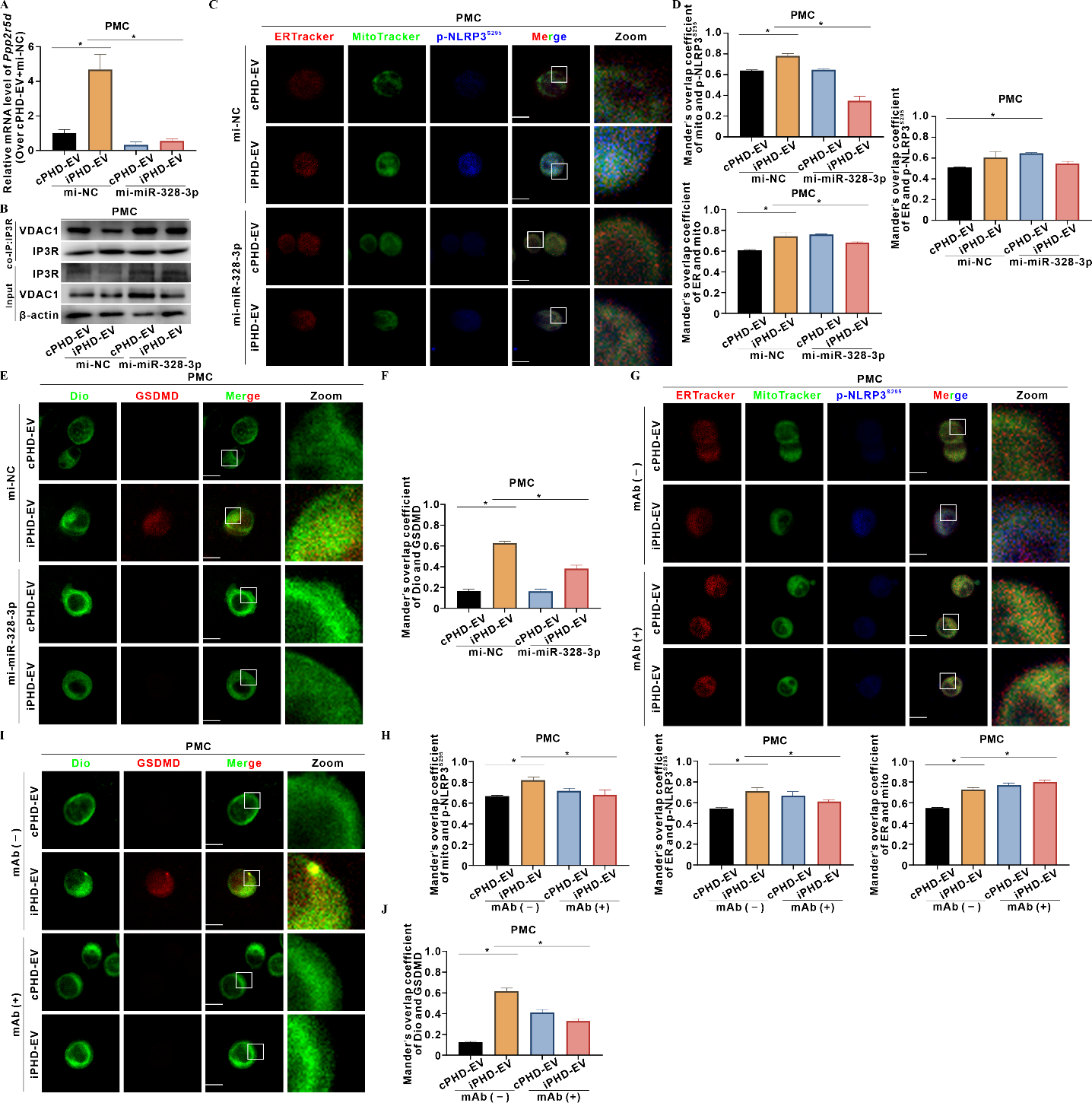


**Figure S6. Targeting miR-328-3p or p-NLRP3^S295^ suppresses MC-LR-induced macrophage metaflammation in primary macrophages.** Primary hepatocytes (PHCs) and hepatic primary macrophages (PMCs) were isolated from C57BL/6J mice; PHCs were used to generate iPHD-EVs or cPHD-EVs for PMC treatment. **(A-F)** PMCs were transfected with mi-miR-328-3p (0.05 μM) or in-miR-328-3p (0.1 μM) for 24 h to generate high- or low-miR-328-3p expression macrophages, using mi-NC or in-NC as controls. **(A)** Relative *Ppp2r5d* mRNA levels were quantified by qRT-PCR. *N* = 3. **(B)** IP3R-VDAC1 interaction was assessed by co-IP. **(C-D)** Representative IF images showing MAM localization of p-NLRP3^S295^ (Blue); mitochondria and ER are labeled with MitoTracker (Green) and ERTracker (Red) (C). Scale bar, 10 μm. Mander's overlap coefficients are presented in bar graphs (D). **(E-F)** Representative IF images showing GSDMD (Red) co-localization with Dio-stained membranes (Green) (E). Scale bar, 10 μm. Mander's overlap coefficients are presented in the bar graph (F). **(G-J)** PMCs were treated with cPHD-EV or iPHD-EV. Anti-p-NLRP3^S295^ mAb conjugated to eTAT (100 μg/ml, 24 h) was applied as an intervention. **(G-H)** Representative IF images showing MAM localization of p-NLRP3^S295^ (Blue); mitochondria and ER were labeled with MitoTracker (Green) and ERTracker (Red) (G). Scale bar, 10 μm. Mander's overlap coefficients are presented in bar graphs (H). **(I-J)** Representative IF images showing GSDMD (Red) co-localization with Dio-stained membranes (Green) (I). Scale bar, 10 μm. Mander's overlap coefficients are presented in the bar graph (J). Data are presented as mean ± SD. *, *P* < 0.05 compared to the control or corresponding group.


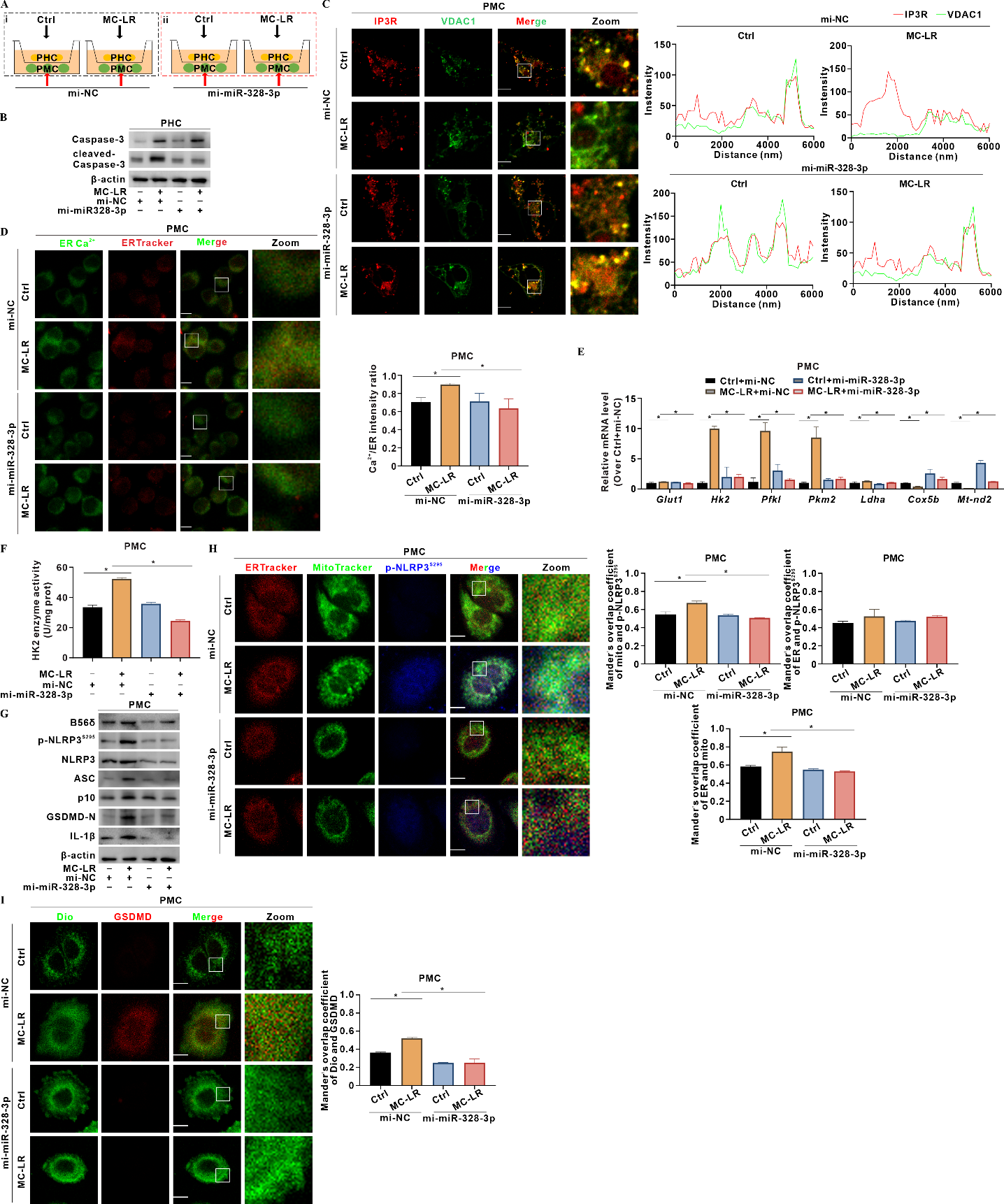


**Figure S7. miR-328-3p overexpression suppresses MC-LR-induced macrophage metaflammation in an *ex vivo* PHC/PMC co-culture model.** Primary hepatocytes (PHCs) and hepatic primary macrophages (PMCs) were isolated from C57BL/6J mice and used in a Transwell co-culture system. PHCs were exposed to MC-LR (0.05 μM, 24 h) to mimic an *in vitro* MASLD microenvironment. PMCs were pre-transfected with miR-328-3p mimic (mi-miR-328-3p, 0.05 μM) or mi-NC (negative control) for 24 h before co-culture analysis. **(A)** Schematic diagram of the *ex vivo* miR-328-3p overexpression intervention in co-cultured PMCs. **(B)** Levels of apoptosis-related proteins were detected by WB. **(C)** Representative IF images showing IP3R (Red) and VDAC1 (Green) co-localization (Left). Scale bar, 10 μm. Immunofluorescence emission spectra for IP3R**–**VDAC1 co-localization are presented (Right). **(D)** Representative IF images showing the level of ER Ca^2+^ (Fluo-5N AM, Green); ER was labeled with ERTracker (Red) staining (Left). Scale bar, 10 μm. Quantification of Ca^2+^/ER fluorescent intensity ratios is presented in the bar graph (Right). **(E)** Relative mRNA levels of glycolysis and oxidative phosphorylation genes were quantified by qRT-PCR. *N* = 3. **(F)** HK2 enzymatic activity was measured. *N* = 3. **(G)** Levels of B56δ, p-NLRP3^S295^, and inflammasome proteins were detected by WB. **(H)** Representative IF images showing MAM localization of p-NLRP3^S295^ (Blue); mitochondrial and ER were labeled with MitoTracker (Green) and ERTracker (Red) (Left). Scale bar, 10 μm. Mander's overlap coefficients are presented in bar graphs (Right). **(I)** Representative IF images showing GSDMD (Red) co-localization with Dio-stained membranes (Green) (Left). Scale bar, 10 μm. Mander's overlap coefficients are presented in a bar graph (Right). Data are presented as mean ± SD. *, *P* < 0.05, compared to the control or corresponding group.

**
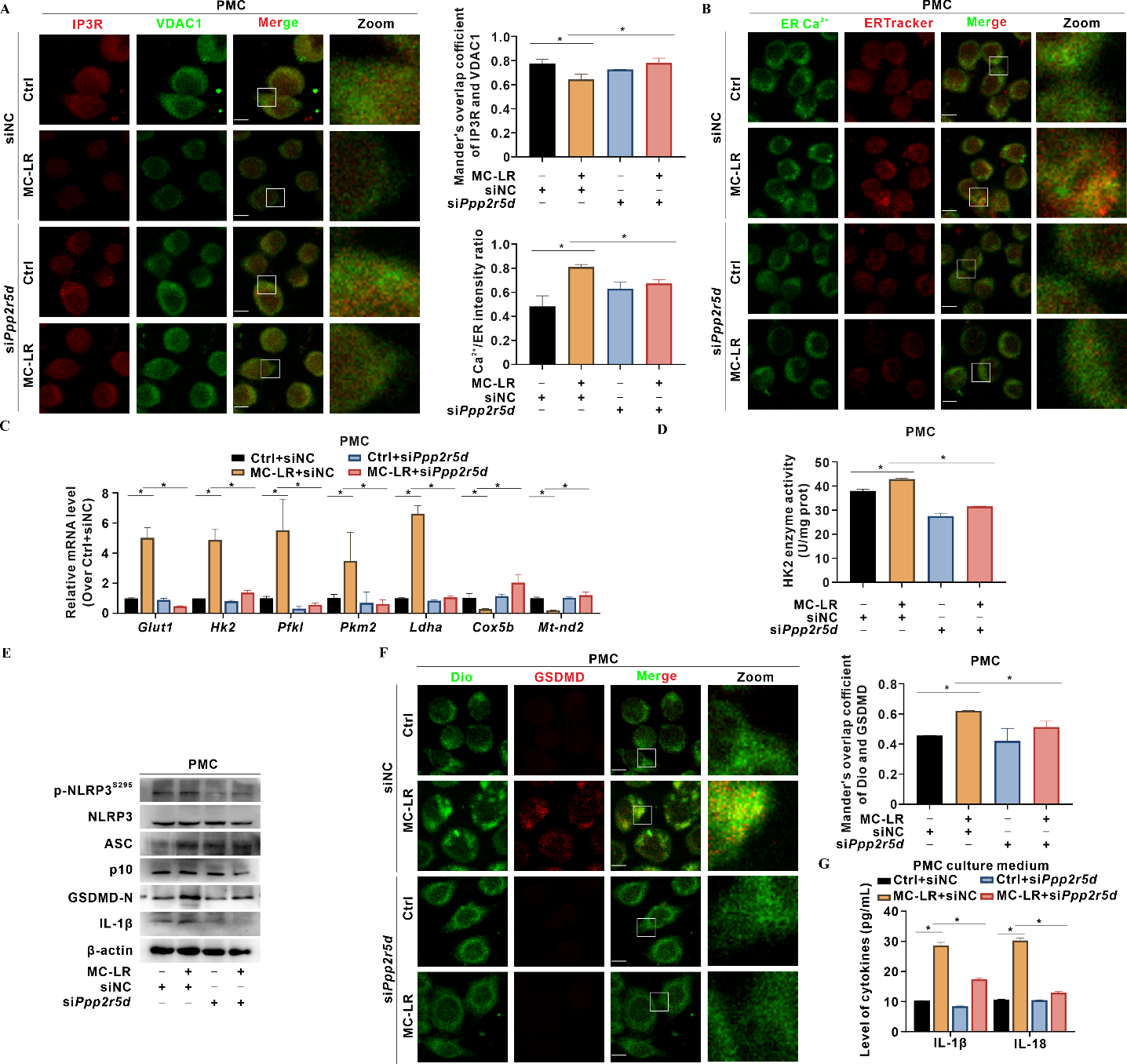
**

**Figure S8. Targeting PP2A-B56δ suppresses MC-LR-induced macrophage metaflammation in an *ex vivo* PHC/PMC co-culture model.** Primary hepatocytes (PHCs) and hepatic primary macrophages (PMCs) were isolated from C57BL/6J mice and used in a co-culture system. PHCs were exposed to MC-LR (0.05 μM, 24 h); PMCs were transfected with si*Ppp2r5d* (0.05 μM) to knock down B56δ or with siNC (negative control) for 24 h before co-culture analysis. **(A)** Representative IF images showing IP3R (Red) and VDAC1 (Green) co-localization (Left). Scale bar, 10 μm. Manders' overlap coefficients are presented in the bar graph (Right). **(B)** Representative IF images showing the level of ER Ca^2+^ (Fluo-5N AM, Green); ER was labeled ER-Tracker (Red) (Right). Scale bar, 10 μm. Quantification of Ca^2+^/ER fluorescent intensity ratios are presented in the bar graph (Left). **(C)** Relative mRNA levels of glycolysis and oxidative phosphorylation genes were quantified by qRT-PCR. *N* = 3. **(D)** HK2 enzymatic activity was measured. *N* = 3. **(E)** Levels of p-NLRP3^S295^ and inflammasome proteins were detected by WB. **(F)** Representative IF images showing GSDMD (Red) co-localization with Dio-labeled plasma membrane (Green) (Left). Scale bar, 10 μm. Manders' overlap coefficients are presented in the bar graph (Right). **(G)** IL-1β and IL-18 levels in culture medium were quantified by ELISA. *N* =3. Data are presented as mean ± SD. *, *P* < 0.05, compared to the control or corresponding group.

**
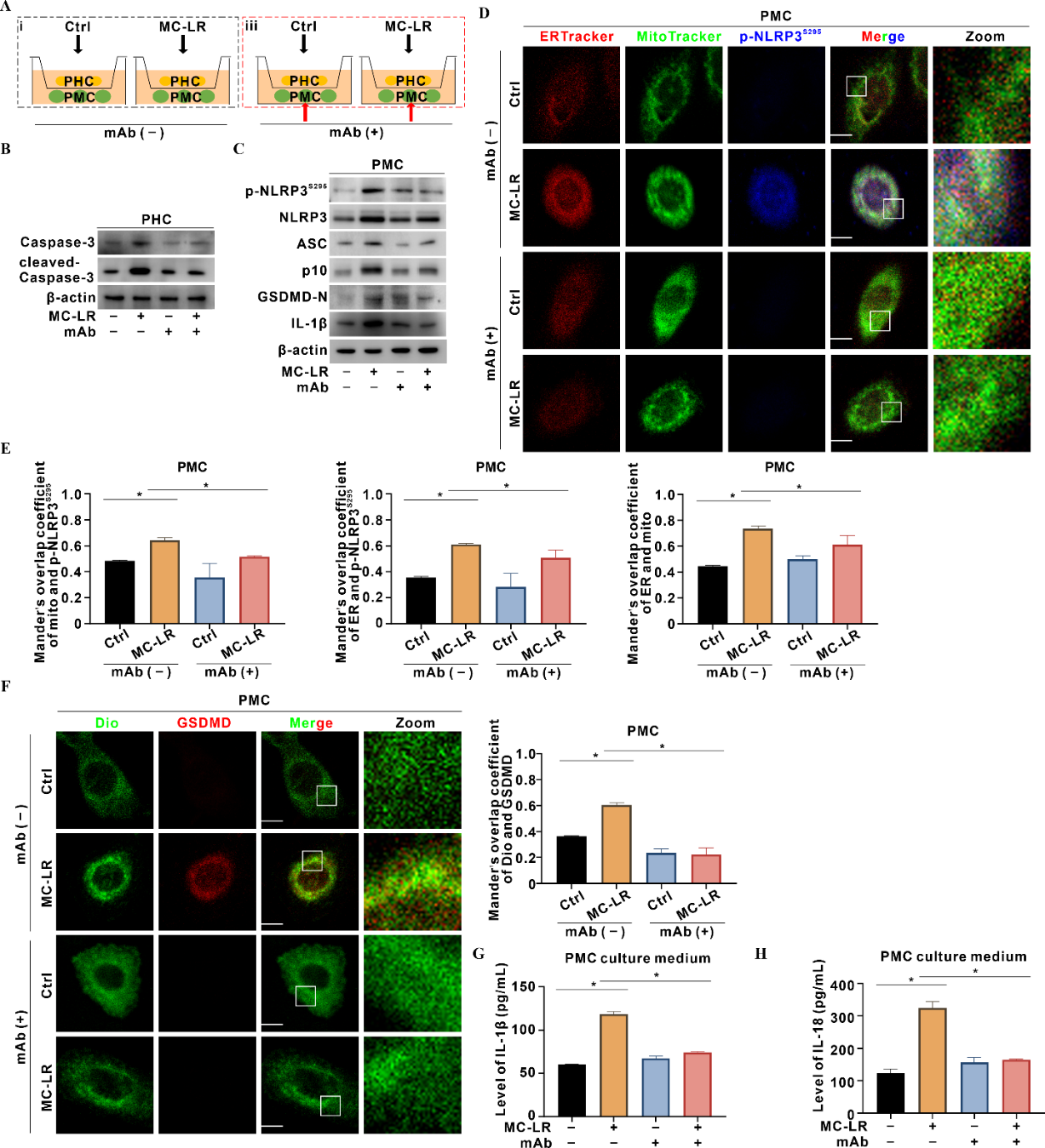
**

**Figure S9. Anti-p-NLRP3^S295^ mAb suppresses MC-LR-induced macrophage p-NLRP3^S295^-dependent metaflammation in an *ex vivo* PHC/PMC co-culture model.** Primary hepatocytes (PHCs) and hepatic primary macrophages (PMCs) were isolated from C57BL/6J mice and used in a co-culture system. PHCs were exposed to MC-LR (0.05 μM, 24 h); PMCs were treated with anti-p-NLRP3^S295^ mAb delivered by eTAT (100 μg/ml, referred to as mAb) for 24 h before co-culture analysis. **(A)** Schematic diagram of *ex vivo* mAb intervention in co-cultured PMCs. **(B)** Levels of apoptosis-related proteins in co-cultured PHCs were detected by WB. **(C)** Levels of p-NLRP3^S295^ and inflammasome proteins in co-cultured PMC were detected by WB. **(D-E)** Representative IF images showing MAM localization of p-NLRP3^S295^ (Blue); mitochondria and ER were labeled with MitoTracker (Green) and ERTracker (Red) (D). Scale bar, 10 μm. Mander's overlap coefficients are presented in bar graphs (E). **(F)** Representative IF images showing GSDMD (Red) co-localization with Dio-stained membranes (Green) (Left). Scale bar, 10 μm. Mander's overlap coefficients are presented in a bar graph (Right). **(G-H)** IL-1β (G) and IL-18 (H) levels in culture medium was quantified by ELISA. *N* = 3. Data are presented as mean ± SD. *, *P* < 0.05 compared to the control or corresponding group.

**
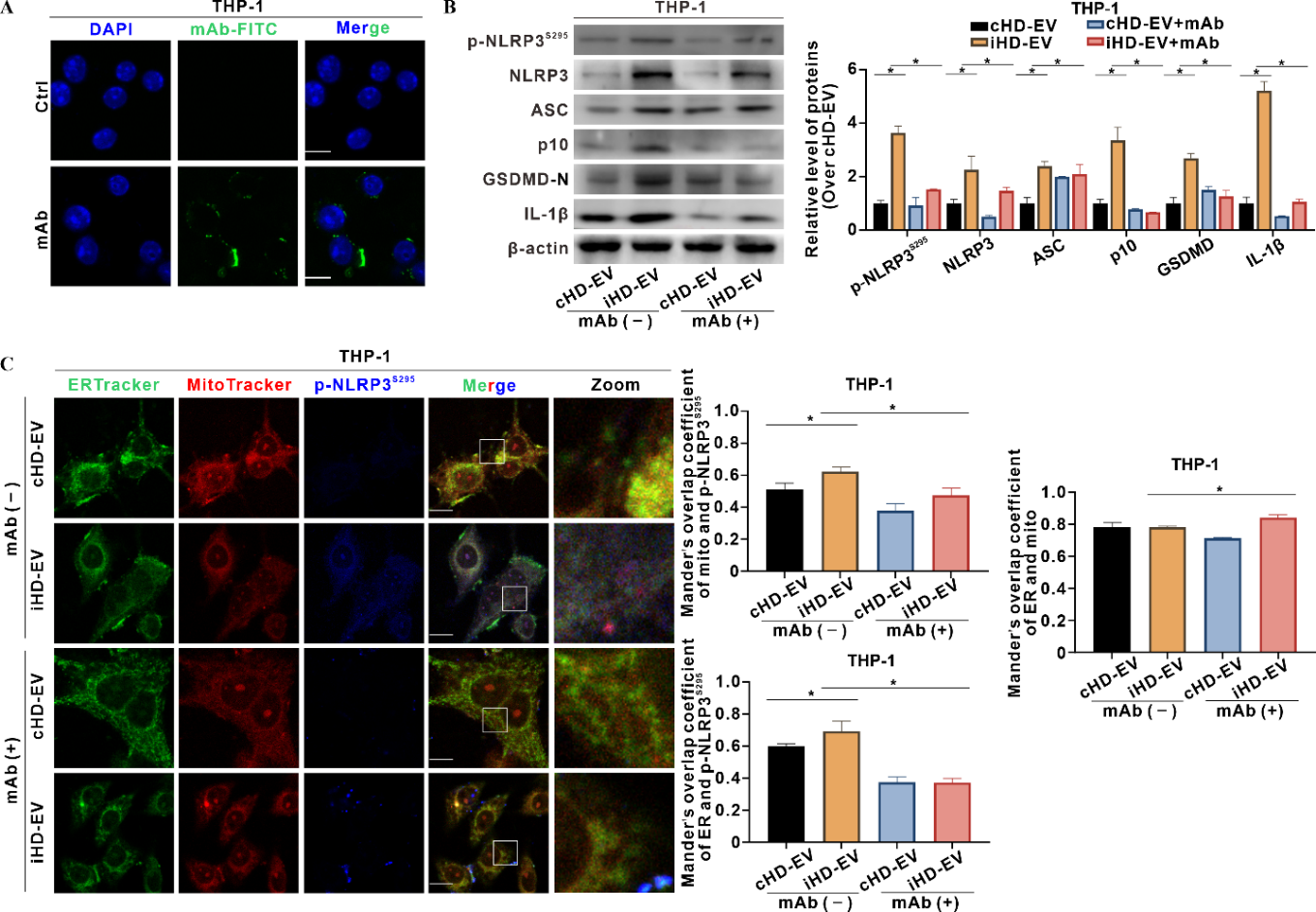
**

**Figure S10. Anti-p-NLRP3^S295^ mAb suppresses iHD-EV-mediated p-NLRP3^S295^-dependent macrophage metaflammation.** THP-1 cells were treated with cHD-EV or iHD-EV (40 μg/mL, 4 h). **(A)** Representative IF images showing anti-p-NLRP3^S295^ mAb (conjugated to FITC, Green) internalized via the eTAT delivery system. Scale bar, 10 μm. **(B)** Levels of p-NLRP3^S295^ and inflammasome proteins were detected by WB (Left); relative expression levels are quantified in the bar graph (Right). **(C)** Representative IF images showing MAM localization of p-NLRP3^S295^ (Blue) (Left). Scale bar, 10 μm. The Mander's overlap coefficients are presented in bar graphs (Right). Data are presented as mean ± SD. *, *P* < 0.05 compared to the control or corresponding group.


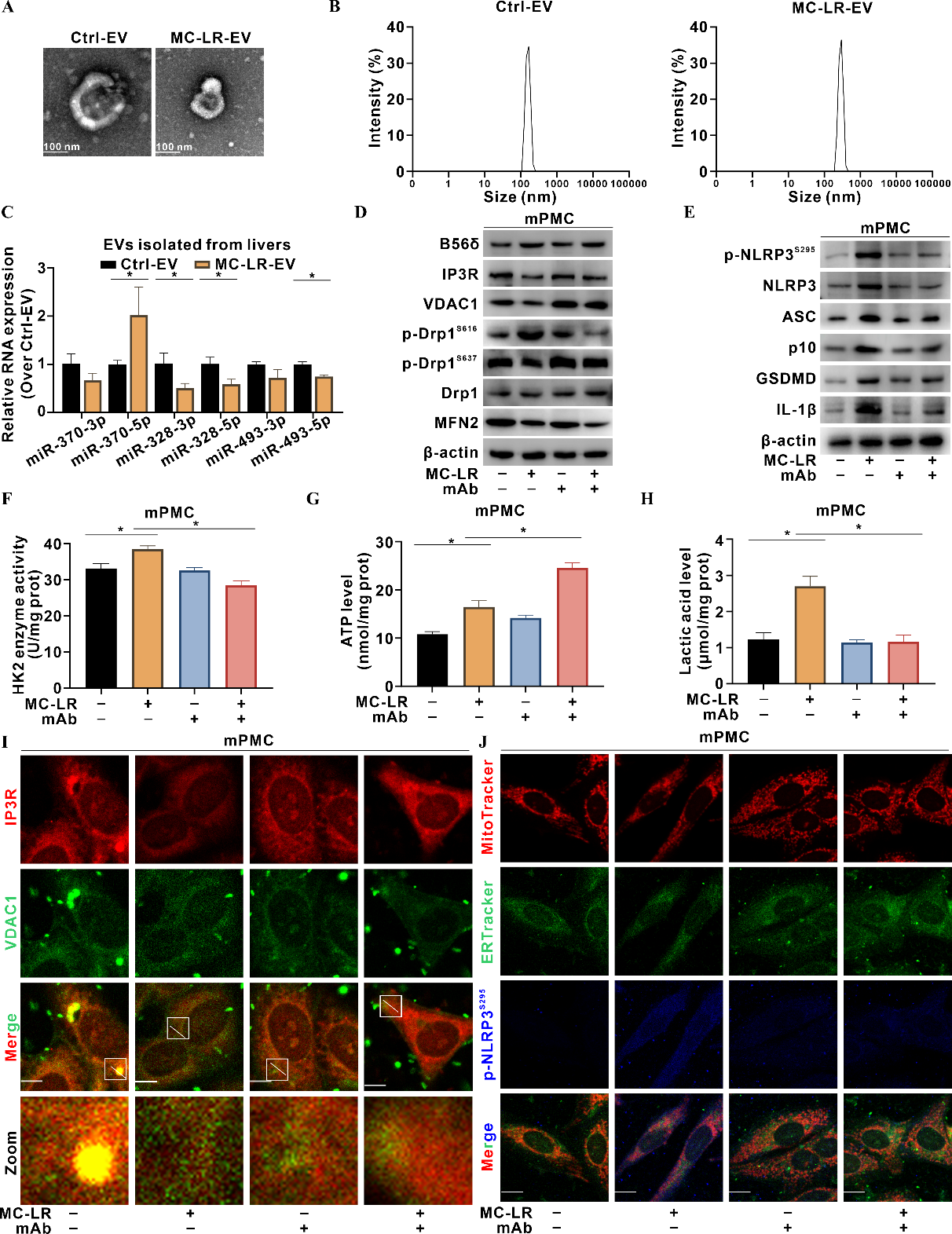


**Figure S11. *In vivo* miR-328-3p/B56δ axis regulates MC-LR-induced hepatic glycolytic reprogramming and p-NLRP3^S295^-dependent metaflammation.** C57BL/6J mice were randomly divided into four groups as illustrated in Figure 7A: control (Ctrl), MC-LR (100 μg/kg), anti-p-NLRP3^S295^ mAb (10 mg/kg), and MC-LR + mAb. *N* = 6 per group. Liver-derived EVs were isolated for analysis. **(A)** Representative TEM images showing the morphology of hepatic Ctrl-EV (Left) and MC-LR-EV (Right). Scale bar, 100 nm. **(B)** Nanoparticle tracking analysis of size distributions for hepatic Ctrl-EV (Left) and MC-LR-EV (Right). **(C)** Relative miRNA levels of miR-370-3p, miR-370-5p, miR-328-3p, miR-328-5p, miR-493-3p, and miR-493-5p in mouse liver tissues were quantified by qRT-PCR. *N* = 3. **(D-J)** Mouse primary macrophages (mPMCs) were isolated from each group. **(D)** Levels of B56δ, IP3R, VDAC1, and MQC-related proteins were detected by WB. **(E)** Levels of p-NLRP3^S295^ and inflammasome proteins were detected by WB. **(F)** HK2 enzymatic activity was measured. *N* = 3. **(G-H)** ATP (G) and lactic acid (H) levels were determined. *N* = 3. **(I)** Representative IF images showing IP3R (Red) and VDAC1 (Green) co-localization. Scale bar, 5 μm. **(J)** Representative IF images showing MAM localization of p-NLRP3^S295^ (Blue); mitochondria and ER are labeled with MitoTracker (Red) and ERTracker (Green). Scale bar, 10 μm. Data are presented as mean ± SD. *, *P* < 0.05 compared to the control or corresponding group.


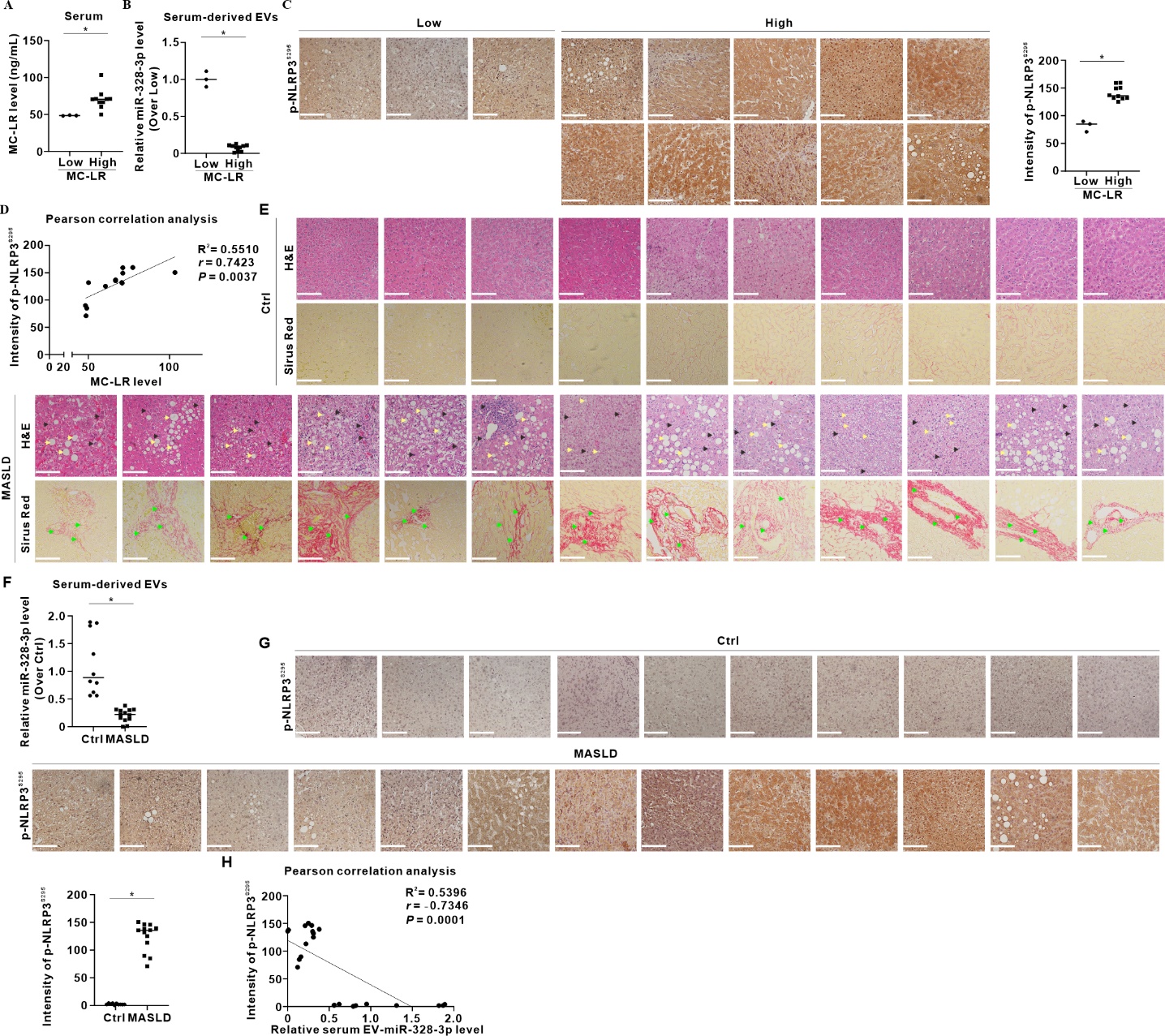


**Figure S12. Clinical correlation between serum EV-miR-328-3p and hepatic p-NLRP3^S295^ expression in MASLD patients stratified by serum MC-LR levels.** Serum and paired liver samples were collected from newly enrolled age- and sex-matched control (Ctrl, *N* = 10) and histologically-confirmed MASLD patients (*N* = 13). **(A-D)** Serum MC-LR levels were quantified; MASLD patients were stratified into MC-LR low-exposure (< 50 ng/L, *N* = 3) or high-exposure (**≥**50 ng/L, *N* = 10) groups. **(B)** Relative miR-328-3p levels in serum-derived EVs were quantified by qRT-PCR. **(C)** Representative IHC images showing hepatic p-NLRP3^S295^expression and sublobular distribution (Left). Scale bar, 50 μm. Quantification of p-NLRP3^S295^ signal intensity per field is presented in a scatter plot (Right). **(D)** Pearson correction analysis between serum MC-LR levels and hepatic p-NLRP3^S295^ intensities across all samples (r = 0.7423, *P* < 0.05). **(E)** Representative H&E (Upper) and Sirius red (Lower) staining images showing hepatic steatosis (yellow arrow), inflammatory infiltration (black arrow), and perisinusoidal fibrosis (green arrow). Scale bar, 50 μm. **(F)** Relative serum EV-miR-328-3p levels were quantified by qRT-PCR. **(G)** Representative IHC images showing hepatic p-NLRP3^S295^ expression and sublobular distribution (Upper). Scale bar, 50 μm. Relative p-NLRP3^S295^ intensities are presented in a scatter plot (Lower). **(H)** Pearson correction analysis between relative serum EV-miR-328-3p and hepatic p-NLRP3^S295^ intensities across all subjects. Data are presented as scatter-dot plots. *, *P* < 0.05, compared to the control group.

**Supplementary reference**

[S1] J. S. Wu, Y. Q. He, Y. Y. Wei, X. Y. Ma, X. Y. Zhang, J. He, L. L. Wang, J. X. He, Y. Han, Z. N. Lin, Y. C. Lin. *Int J Biol Macromol* **2025**, 308, 142696.

[S2] L. Che, C. L. Yang, Y Chen, Z. L. Wu, Z. B. Du, J. S. Wu, C. L. Gan, S. P. Yan, J. Huang, N. J. Guo, Y. C. Lin, Z. N. Lin. *Chemosphere* **2021**; 262:127878.
